# Association of circulating PLA2G7 levels with cancer cachexia and assessment of darapladib as a therapy

**DOI:** 10.1002/jcsm.12758

**Published:** 2021-08-23

**Authors:** Pauline Morigny, Doris Kaltenecker, Julia Zuber, Juliano Machado, Lisa Mehr, Foivos‐Filippos Tsokanos, Hanna Kuzi, Chris D. Hermann, Michael Voelkl, German Monogarov, Christoph Springfeld, Victor Laurent, Bernd Engelmann, Helmut Friess, Inka Zörnig, Achim Krüger, Jeroen Krijgsveld, Olga Prokopchuk, Søren Fisker Schmidt, Maria Rohm, Stephan Herzig, Mauricio Berriel Diaz

**Affiliations:** ^1^ Institute for Diabetes and Cancer Helmholtz Center Munich Neuherberg Germany; ^2^ Joint Heidelberg‐IDC Translational Diabetes Program, Inner Medicine 1 Heidelberg University Hospital Heidelberg Germany; ^3^ German Center for Diabetes Research (DZD) Neuherberg Germany; ^4^ Department of Surgery, Klinikum rechts der Isar, School of Medicine Technical University of Munich Munich Germany; ^5^ School of Medicine, Institutes of Molecular Immunology and Experimental Oncology Technical University of Munich Munich Germany; ^6^ Institute of Laboratory Medicine University Hospital Ludwig‐Maximilian University Munich Germany; ^7^ German Cancer Research Center (DKFZ) Heidelberg Germany; ^8^ Department of Medical Oncology, National Center for Tumor Diseases and Internal Medicine VI Heidelberg University Hospital Heidelberg Germany; ^9^ Medical Faculty Heidelberg University Heidelberg Germany; ^10^ Chair Molecular Metabolic Control Technical University of Munich Munich Germany

**Keywords:** Cancer cachexia, PLA2G7, Mouse models, Cancer patients, Darapladib, Biomarker

## Abstract

**Background:**

Cancer cachexia (CCx) is a multifactorial wasting disorder characterized by involuntary loss of body weight that affects many cancer patients and implies a poor prognosis, reducing both tolerance to and efficiency of anticancer therapies. Actual challenges in management of CCx remain in the identification of tumour‐derived and host‐derived mediators involved in systemic inflammation and tissue wasting and in the discovery of biomarkers that would allow for an earlier and personalized care of cancer patients. The aim of this study was to identify new markers of CCx across different species and tumour entities.

**Methods:**

Quantitative secretome analysis was performed to identify specific factors characteristic of cachexia‐inducing cancer cell lines. To establish the subsequently identified phospholipase PLA2G7 as a marker of CCx, plasma PLA2G7 activity and/or protein levels were measured in well‐established mouse models of CCx and in different cohorts of weight‐stable and weight‐losing cancer patients with different tumour entities. Genetic PLA2G7 knock‐down in tumours and pharmacological treatment using the well‐studied PLA2G7 inhibitor darapladib were performed to assess its implication in the pathogenesis of CCx in C26 tumour‐bearing mice.

**Results:**

High expression and secretion of PLA2G7 were hallmarks of cachexia‐inducing cancer cell lines. Circulating PLA2G7 activity was increased in different mouse models of CCx with various tumour entities and was associated with the severity of body wasting. Circulating PLA2G7 levels gradually rose during cachexia development. Genetic PLA2G7 knock‐down in C26 tumours only partially reduced plasma PLA2G7 levels, suggesting that the host is also an important contributor. Chronic treatment with darapladib was not sufficient to counteract inflammation and tissue wasting despite a strong inhibition of the circulating PLA2G7 activity. Importantly, PLA2G7 levels were also increased in colorectal and pancreatic cancer patients with CCx.

**Conclusions:**

Overall, our data show that despite no immediate pathogenic role, at least when targeted as a single entity, PLA2G7 is a consistent marker of CCx in both mice and humans. The early increase in circulating PLA2G7 levels in pre‐cachectic mice supports future prospective studies to assess its potential as biomarker for cancer patients.

## Introduction

Cancer cachexia (CCx) is a multifactorial wasting disorder characterized by unintentional loss of body weight and functional impairment that reduces the quality of life, the tolerance and responsiveness to anticancer therapies, and the overall survival of cancer patients.^1^, ^2^ This syndrome is notably defined by an ongoing loss of skeletal muscle mass, with or without loss of fat mass, which may predispose patients to cardiorespiratory complications (cardiac arrhythmias and respiratory difficulties due to diaphragm muscle weakness).^1^ CCx is observed in many cancer patients, affecting even up to 85% in some cancer types, and importantly contributes to cancer‐related morbidity and mortality.^3^, ^4^, ^5^, ^6^ It is particularly recurrent in some malignancies such as pancreatic, oesophageal, colorectal, lung, head and neck, and haematological cancers, and cachexia prevalence is higher in advanced stages of the disease.^1^

Mechanistically, CCx is a multi‐organ syndrome, which leads to an imbalance between energy intake and expenditure that cannot be fully rescued by nutritional approaches. Common features include anorexia, proteolysis in cardiac and skeletal muscles, lipolysis and energy expenditure in adipose tissues, acute‐phase response and futile cycles in the liver, systemic inflammation, and inhibition of anabolic pathways.^1^, ^3^, ^7^, ^8^ Previous studies focusing on the identification of intra‐organ and inter‐organ mechanisms responsible for tissue wasting have identified some tumour‐derived and host‐derived factors as cachexia mediators, notably including pro‐inflammatory factors such as interleukin‐6 (IL‐6), tumour necrosis factor‐α, interleukin‐1β, or growth differentiation factor 15 (GDF‐15).^1^ However, none of them has led to efficient routine therapy so far, and there is currently no defined standard of care for cachectic cancer patients.

Cancer cachexia can evolve from pre‐cachexia to refractory cachexia; however, not every patient will progress to the most advanced stages of the disease.^2^ Treatment of patients with refractory cachexia may be ineffective and inappropriately invasive, and recent clinical trials now comprise patients with less advanced stages of the disease.^9^, ^10^ The future of anticachexia therapies includes an earlier management of the disease, ideally at a pre‐cachectic stage, and a personalized care of the patients.^1^ However, a main challenge remains in the lack of reliable biomarkers for diagnosis of pre‐cachexia and efficient classification of cancer patients.

Here, we identified the phospholipase A2 group VII (PLA2G7) as a tumour‐derived and host‐derived factor specifically increased in both well‐established mouse models of CCx and cachectic cancer patients. High PLA2G7 expression and secretion were hallmarks of cachexia‐inducing cancer cell lines, and elevated circulating levels of PLA2G7 were a robust marker of CCx in different types of malignancies such as colorectal, lung, and pancreatic cancers. PLA2G7 is an enzyme reported to harbour both anti‐inflammatory and pro‐inflammatory functions, through the hydrolysis of platelet‐activating factor (PAF) and short‐chain/oxidized phospholipids, respectively,^11^, ^12^ and its inhibition has been tested in various clinical trials for different inflammatory diseases.^13^, ^14^, ^15^, ^16^ We therefore assessed the therapeutic potential of the specific PLA2G7 inhibitor darapladib in preventing CCx development in pre‐cachectic tumour‐bearing mice (i.e. before onset of body weight loss). Specific pharmacological inhibition of PLA2G7 alone was not sufficient to counteract body wasting and systemic inflammation. Importantly, this study highlights the predictive potential of PLA2G7 in different tumour entities and may pave the way for future studies on PLA2G7 as biomarker of CCx.

## Materials and methods

### Cell culture

MC38, NC26, Panc02, C26, Lewis lung cancer (LLC), 8025, and HEK293T cells were grown in high‐glucose Dulbecco's modified Eagle's medium (DMEM) with pyruvate (Life Technologies #41966052), supplemented with 10% fetal bovine serum (FBS, Sigma‐Aldrich #F7524) and 1% penicillin–streptomycin (Thermo Fisher #15140122). Cells were seeded at a density of 13 500 cells/cm^2^ and trypsinized (Thermo Fisher #25300054) every 2–3 days, once they reached 80% confluence. MC38, NC26 and C26 are murine colon carcinoma cell lines; Panc02 and 8025 are murine pancreatic carcinoma cell lines; LLC is a murine lung cancer cell line.

3T3‐L1 cells (ATCC #CL‐173) were seeded at a density of 6250 cells/cm^2^ and grown in high‐glucose DMEM with pyruvate supplemented with 10% FBS and 1% penicillin–streptomycin for 4 days until they reached 100% confluence. At Day 0, differentiation was initiated by adding 1 μg/mL insulin (Sigma‐Aldrich #I9278), 0.25 μM dexamethasone (Sigma‐Aldrich #D4902), 0.5 mM 3‐isobutyl‐1‐methylxanthine (IBMX, Sigma‐Aldrich #I7018), and 2 μM rosiglitazone (Sigma‐Aldrich, #R2408) to the media. Dexamethasone, IBMX, and rosiglitazone were removed from the media after 2 days and insulin after 4 days of differentiation. The cells were differentiated for four extra days in normal media before use.

C2C12 cells (ATCC #CRL‐1772) were seeded at a density of 5000 cells/cm^2^ and grown in high‐glucose DMEM with pyruvate supplemented with 10% FBS and 1% penicillin–streptomycin until they reached 80–100% confluence. Media were then switched to 1% FBS for 4 days to promote myotube differentiation. After 4 days, long contractile myotubes were used for experiments.

Cell lines were regularly tested for mycoplasma contamination by polymerase chain reaction (PCR) according to manufacturer's instructions (Promokine, #PK‐CA91–1048).

#### Quantitative secretome analysis

60–70% confluent C26 and MC38 cells, grown in normal media, were switched to SILAC medium [DMEM (non‐GMP formulation without Met, Arg, and Lys), 10% FBS (dialyzed), 4 mM l‐glutamine, and 100 mg/L primocin] for 30 min. Media were then replaced by SILAC medium supplemented with 0.1 mM l‐azidohomoalanine (AHA) and either 84 mg/mL [^13^C_6_, ^15^N_4_] l‐arginine and 146 mg/mL [^13^C_6_, ^15^N_2_] l‐lysine (heavy label) or 84 mg/mL [^13^C_6_] l‐arginine and 146 mg/mL [4,4,5,5‐D_4_] l‐lysine (intermediate label) for 24 h. Conditioned media (CM) enriched with newly synthesized proteins were collected, centrifuged to remove debris, supplemented with protease inhibitor (Roche), and frozen at −80°C until further processing. Sample preparation for mass spectrometry, liquid chromatography–tandem mass spectrometry analysis, data processing, and statistical analysis were performed as previously described.^17^ Data represent the mean of three independent biological replicates.

#### Generation of C26‐sh*Pla2g7* stable cell line

70% confluent HEK293T cells were transfected with plasmids expressing either a control shRNA or a shRNA targeting *Pla2g7* (Sigma‐Aldrich, Mission shRNA DNA clone pLKO.1 #SHCLND‐NM_013737) together with lentivirus packaging plasmids (Addgene psPax2 #12260, pMD2.G #12259 provided by Didier Trono) previously combined to an OptiMEM (Life Technologies #31985062)/lipofectamine (Thermo Scientific #11668019) mixture. The day after, cells were switched to normal media supplemented with 2% fatty acid free bovine serum albumin (Sigma‐Aldrich #A7030). Media containing lentivirus were collected 24 h later, filtered with 0.45 μm filters, and stored at −80°C until use.

50% confluent C26 cells were incubated for 24 h with lentivirus carrying either the control shRNA or the shRNA targeting *Pla2g7*. Transduced cells were selected using 3.5 μM puromycin (Thermo Fisher #A1113803) for 3 days. Once all non‐transduced cells had died, puromycin‐resistant cells were trypsinized and expanded in normal media. Growth rate of control C26 cells (C26‐shCTR) and C26 cells lacking PLA2G7 (C26‐sh*Pla2g7*) was estimated by comparing the number of cells during trypsination with the number of cells initially seeded.

#### Production of conditioned media and treatment of target cells

For treatment of adipocytes, cancer cells were grown in high‐glucose DMEM with pyruvate, 10% FBS, and 1% penicillin–streptomycin for 3 days, until they reached 80% confluence. For treatment of myotubes, 60–70% confluent cancer cells were switched into 1% FBS and 1% penicillin–streptomycin media for 24 h. CM were then collected, centrifuged at 300 *x* *g*, 4°C for 10 min to remove cells and at 2000 *x* *g*, 4°C for 10 min to remove cell debris. CM were either used directly or stored at −80°C until use. Cancer cells used to produce CM were counted to ensure a similar number between the different cell lines compared. CM were diluted with fresh media (1:1, v/v for adipocytes; 3:1, v/v for myotubes) before treatment for 48 h. For darapladib treatment, dimethylsulfoxid (DMSO, vehicle) and 1 μM darapladib (Biorbyt #ORB181231) resuspended in DMSO were freshly added to control or C26 CM before treatment of adipocytes and myotubes. CM were renewed every 24 h.

#### Measurement of glycerol release

After 48 h of treatment with CM, glycerol released into the medium by adipocytes was measured using a commercially available kit (Sigma‐Aldrich #F6428). Data were normalized to initial glycerol content in CM and to protein content of adipocytes present in the well to account for cell number. Experiment was repeated using different independent cultures of adipocytes.

#### Quantification of myotube diameters

After 48 h of treatment with CM, approximately 10 pictures of myotubes per well were recorded (×40 objective, #Nikon Eclipse Ts2). Approximately 100 myotubes were quantified per well using ImageJ software, and the average was considered as *n* = 1 independent biological replicate. Experiment was repeated using different independent cultures of myotubes.

### Animal experiments

*In vivo* experiments were carried out using 10‐ to 12‐week‐old male BALB/c or C57BL/6J mice that were obtained from Charles River Laboratories (CRL, Brussels, Belgium). All mice were maintained under specific pathogen‐free conditions on a 12 h light–dark cycle at 22°C with *ad libitum* access to regular rodent chow diet (Kliba Nafag #3437, Promivi Kliba AG, Kaiseraugst, Switzerland) and water. In each animal experiment, mice were assigned to groups in a manner that body weight, and lean and fat mass were similar between the groups as confirmed by non‐significant statistical analysis. Animal handling and experimentation were performed in accordance with the institutional animal welfare officer, and the necessary licences were obtained from the state ethics committee and government of Upper Bavaria (Nos. 55.2‐2532.Vet_02‐16‐136 and ROB‐55.2‐2532.Vet_02‐18‐93).

In different experiments, mice were injected subcutaneously into the right flank with 0.5 million of MC38, 2 million of LLC, 1 million of NC26, or 1 million of C26 cells, resuspended in Dulbecco's phosphate‐buffered saline (PBS, Thermo Fisher #14190250). Non‐tumour‐bearing healthy control mice were injected with PBS. For the experiment including the PLA2G7 inhibitor treatment, mice received 50 mg/kg of darapladib (Biorbyt #ORB181231) or a DMSO/carboxymethylcellulose 0.5% solution (10:90, v/v, 50 μL per 10 g body weight) as vehicle control daily by oral gavage once tumours were palpable. For the longitudinal prospective study involving PBS, NC26, and C26 tumour‐bearing mice, approximately 20 μL of blood was withdrawn from the tail vein throughout CCx development. Mice were monitored for 2–4 weeks after cell implantation, and tumour growth, body weight, food, and water intakes were recorded daily. Mice were sacrificed by an overdose of ketamine/xylazine after having lost 10–15% of their initial body weight or reached humane endpoints (tumour >1.5 cm or ulceration of tumour). In the C26‐sh*Pla2g7* and darapladib experiments, all remaining mice were sacrificed once the last control C26‐shCTR or vehicle‐treated mice were sacrificed, respectively. In the C26‐pre‐cachexia vs. C26‐cachexia study, the cachectic C26 tumour‐bearing group was defined by a 10–15% body weight loss, whereas the C26‐pre‐cachectic group was characterized by a similar tumour size but a non‐significant loss of body weight at the moment of sacrifice. A C26‐pre‐cachectic control mouse with a matching tumour size was euthanized on the same day than a cachectic C26 tumour‐bearing mouse. Tumour volumes prior sacrifice were estimated using the formula [*π*/6 * 1.69 * (wide * length)^3/2^]. Body composition was measured by EchoMRI™.

Blood was withdrawn from the vena cava, transferred into EDTA tubes, and centrifuged at 2000 *x* *g*, 4°C for 10 min. Plasma was immediately aliquoted, snap‐frozen in liquid nitrogen, and stored at −80°C until further processing. Circulating leucocytes were isolated using Histopaque‐1083 (Sigma‐Aldrich #10831) according to manufacturer's instructions within 1 h after blood removal. Isolated leucocytes were lysed in TRIzol (Life Technologies #15596018) for further mRNA analysis. Tumours and organs including inguinal white adipose tissue (iWAT), epididymal WAT (eWAT), brown adipose tissue, heart, gastrocnemius muscle (GC), tibialis anterior (TA), spleen, lymph nodes and liver were collected, snap‐frozen, and stored at −80°C. Plasma levels of total cholesterol, HDL, LDL, glucose, total proteins, AST, and ALT were measured using a Beckman Coulter AU480 Chemistry Analyzer.

The pancreatic cancer transgenic mouse model KPC (Kras+/LSL‐G12D; Trp53+/LSL‐R172H; and Pdx‐1+/Cre) used in this study was described in detail elsewhere.^18^ Breeding of KPC mice was performed in compliance with the Tierschutzgesetz des Freistaates Bayern and approved by the Regierung von Oberbayern. Male mice were employed in this study and kept at the animal facility of Klinikum rechts der Isar (Munich, Germany) at room temperature (21°C) under specific pathogen‐free conditions. All mice were maintained in filter‐topped cages with autoclaved food and water. Median age of tumour‐bearing KPC mice was 14 weeks. Body weight (BW) was measured immediately before mice were sacrificed (BWt0) as well as 1 week prior to death (BWt‐1). Percentage of body weight loss was calculated as ‘percentage of previous body weight’ (BWt0/BWt − 1 × 100). Blood was taken from sacrificed mice using an EDTA‐coated syringe and centrifuged at 500 *x* *g*, 4°C for 10 min. Plasma samples were snap‐frozen in liquid nitrogen and stored at −80°C. After blood collection, iWAT, eWAT, and GC muscles were collected and snap‐frozen in liquid nitrogen, and tissue weight was measured.

### Patient cohorts and samples

#### Cohort 1

Serum samples of colorectal and pancreatic cancer patients were collected in the Department of Medical Oncology at the National Center for Tumor Diseases, Heidelberg University Hospital in Heidelberg, Germany. Patients had given their informed consent under the ethic's vote S‐022/2013 of the local ethics commission of the Medical Faculty at Heidelberg University. The day of blood sampling, body weight loss in the past 3 months (reported by patients themselves), and the current patients' body weights were recorded. Patients with no reported body weight loss in the last 3 months were classified as weight‐stable, and patients exhibiting a body weight loss equal or >5% of their body weight 3 months before were classified as weight‐losing. Given the lack of information on body weight changes in the time preceding 3 months and on the presence of sarcopenia, we did not refer to the definition of Fearon *et al*. of cachexia to classify the patients.^2^ Clinical information of Cohort 1 is provided in *Table* 1.

**Table 1:** Clinical data of Cohort 1 including healthy individuals and weight‐stable and weight‐losing patients with pancreatic or colorectal cancer

	Healthy controls	Weight‐stable patients	Weight‐losing patients
*n*	10	19	10
Gender			
Male	5	13	6
Female	5	6	4
Age	39.2 (±4.7)**	64.2 (±2.2)	63.8 (±3.9)
Tumour entities			
Pancreatic carcinoma		9	6
Colorectal carcinoma		10	4
UICC stage at the time of blood sampling		IV	IV
Body weight at the time of blood sampling (kg)		74.5 (±2.5)	69.4 (±6.5) NS
Body weight loss in the last 3 months (kg)		Reported no weight loss	7.7 (±1.9)
Body weight loss in the last 3 months (%)		Reported no weight loss	9.5 (±1.5)

UICC, Union for International Cancer Control.

Data are mean ± standard error of the mean. Statistical analyses were performed using Kruskal–Wallis with Dunn's *post hoc* tests and unpaired *t*‐test. Tests were two sided.

** *P*< 0.01 versus weight‐stable and weight‐losing patients. NS, non‐significant.

Serum from healthy individuals was obtained from Innovative Research™.

#### Cohort 2

Enrolment of Munich pancreatic cancer patients (Stages I–IV) occurred in the Department of Surgery, Klinikum Rechts der Isar. This study was approved by the Ethics Committee of the Medical Faculty of the Technical University of Munich (Germany; #1946/07 and #409/16S), and written informed consent was obtained from all participants before surgery or before blood sampling. The analysis was conducted on a pseudonymized data set. The study population comprised patients with pancreatic cancer, who underwent oncological treatment (staging or resection) and who agreed to participate in the study. Diagnosis of pancreatic cancer patients was verified by definitive histological examination of retrieved biopsies, or in pancreatic cancer patients without surgery, by cytology or clinical/radiological information to the best of our knowledge. We used the eighth edition of the Union for International Cancer Control tumour–node–metastasis classification and staging system for pancreatic cancer.

Weight was measured at the time of admission to the hospital. Weight histories over the 6 months preceding admission were collected. Cachexia was defined according to Fearon *et al*.^2^ Clinical information of Cohort 2 is provided in *Table* 2.

**Table 2:** Clinical data of Cohort 2 including non‐cachectic (Non‐Cax) and cachectic (Cax) patients with pancreatic cancer (the definition of Fearon *et al*. of cachexia)

	Non‐Cax	Cax
*n*	24	46
Gender		
Male	9	22
Female	15	24
Age	68.4 (±2.6)	69.3 (±1.5) NS
Tumour entities	Pancreatic cancer	Pancreatic cancer
UICC stage		
I	1	0
II	9	22
III	6	8
IV	8	16
Body weight at the time of inclusion in the clinical study (kg)	71.8 (±3.0)	69.1 (±2.1) NS
Body weight loss (kg)	0.8 (±0.3)	10.2 (±1.0)****
Body weight loss (%)	1.0 (±0.3)	12.7 (±1.1)****

UICC, Union for International Cancer Control.

Data are mean ± standard error of the mean. Statistical analyses were performed using unpaired *t*‐test or Mann–Whitney test. Tests were two sided.

**** *P* < 0.0001. NS, non‐significant.

Blood samples were collected, and plasma was obtained within 30 min by centrifugation of whole blood at 1000 *x* *g* for 15 min. Plasma samples were immediately snap‐frozen in liquid nitrogen and stored at −80°C.

### Platelet‐activating factor acetylhydrolase activity measurement

A total of 3–10 μL of concentrated CM (Merck Millipore #UFC501024), mouse, or human plasma were used to measure extracellular PAF acetylhydrolase (PAF‐AH) activity according to manufacturer's instructions (Cayman Chemical #760901). Activity in CM was normalized to the number of cells present in the plate. To assess intracellular PAF‐AH activity, 50–100 mg of tissue were homogenized in ice‐cold buffer (0.1 M Tris–HCl pH 7.2, 500 μL for 50 mg of tissue), centrifuged at 10 000 *x* *g*, 4°C for 15 min, and 10 μL of supernatant was assessed according to manufacturer's instructions (Cayman Chemical #760901).

### Enzyme‐linked immunosorbent assays

Circulating levels of PLA2G7 (human samples), GDF‐15 (human samples), IL‐6 (human and mouse samples), and PAF (mouse samples) were assessed using specific ELISA kits (R&D Systems #DPLG70, #DGD150, #HS600C, and #M6000B; Cusabio #CSB‐E08199m) according to manufacturer's instructions. Dilution of plasma samples for mouse IL‐6 and PAF detection was optimized to 2/5 and 1/6, respectively.

### Real‐time quantitative PCR

**Figure 1:**
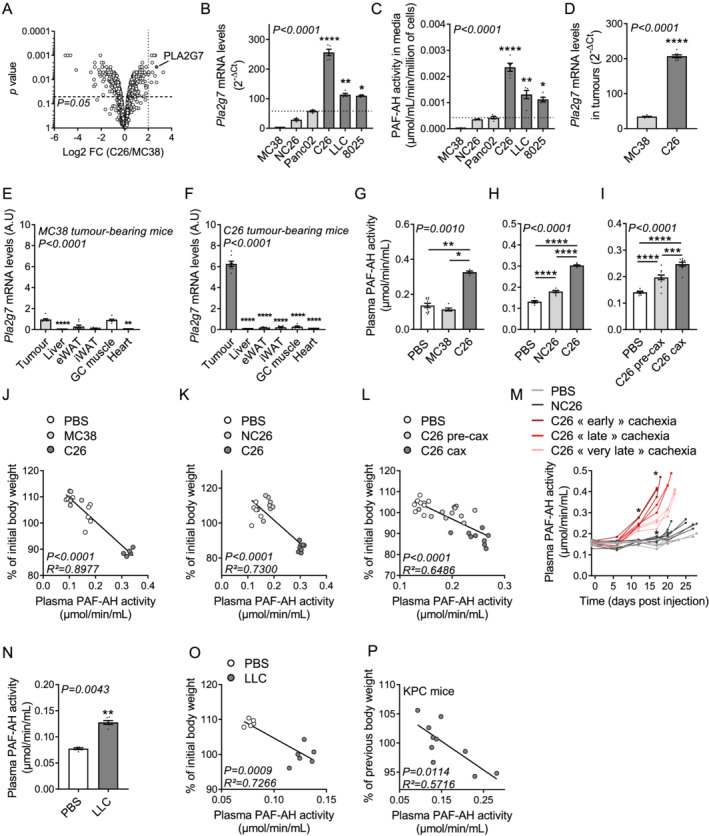
High circulating levels of PLA2G7 are a hallmark of CCx in different mouse models with various tumour entities. *(A)* Proteomic analysis of C26 vs. MC38 conditioned media [*n* = 3 biological replicates per group; data are log_2_ values of fold change (FC) C26/MC38]. C26 is a cachexia‐inducing cancer cell line, while MC38 is a non‐cachexia‐inducing cancer cell line. *(B)* *Pla2g7* mRNA levels (*n* = 5 biological replicates per group) and *(C)* PAF‐AH activity levels in conditioned media (*n* = 5 biological replicates per group) from various cancer cell lines with different cachexia‐inducing properties (light grey bars: no cachexia‐inducing properties; dark grey bars: cachexia‐inducing properties). *(D)* *Pla2g7* mRNA levels in tumours of non‐cachectic MC38 (light grey bar) and cachectic C26 (dark grey bar) tumour‐bearing mice (*n* = 8 animals per group). *(E)* Relative *Pla2g7* mRNA levels in tumours (grey bars) and metabolic tissues (white bars) of non‐cachectic MC38 and *(F)* cachectic C26 tumour‐bearing mice (*n* = 8 animals per group). *(G–I)* Plasma PAF‐AH activity levels in *(G)* PBS (white bar, *n* = 10 animals), non‐cachectic MC38 (light grey bar, *n* = 5 animals), and cachectic C26 (dark grey bar, *n* = 5 animals) tumour‐bearing mice; *(H)* PBS (white bar, *n* = 6 animals), non‐cachectic NC26 (light grey bar, *n* = 7 animals), and cachectic C26 (dark grey bar, *n* = 8 animals) tumour‐bearing mice; and *(I)* PBS (white bar, *n* = 9 animals), pre‐cachectic (C26‐precax, light grey bar, *n* = 11 animals), and cachectic (C26‐cax, dark grey bar, *n* = 9 animals) C26 tumour‐bearing mice. Of note, activity data from MC38 and C26 tumour‐bearing mice are from another cohort than the one presented in *Figures* S1A–S1E but which shared the same properties (in terms of loss of body weight, and fat and muscle mass for a similar tumour size). *(J–L)* Linear regression analyses comparing plasma PAF‐AH activity and loss of body weight (expressed as percentage of initial body weight) in the animal experiments presented in *Figures* 1G–1I. *(M)* Longitudinal prospective study showing plasma PAF‐AH activity in PBS (light grey lines, *n* = 6 animals), non‐cachectic NC26 (dark grey lines, *n* = 7 animals), and cachectic C26 (red lines, *n* = 8 animals) tumour‐bearing mice throughout cachexia development. Final activity was measured on the day of sacrifice. C26 mice were divided into three groups based on their time course of cachexia development including an early (Days 17 and 18, dark red lines), late (Days 20 and 21, bright red lines), and very late (Day 22, light red lines) cachexia development. The graph shows individual mice data. Statistical analysis was performed using a paired two‐way ANOVA with Bonferroni *post hoc* tests until Day 17 where all the mice were still included. *(N, O)* Plasma PAF‐AH activity *(N)* and linear regression analysis comparing plasma PAF‐AH activity and loss of body weight *(O)* in PBS (white bar/dots, *n* = 5 animals) and LLC tumour‐bearing mice (dark grey bar/dots, *n* = 6 animals). *(P)* Linear regression analysis comparing plasma PAF‐AH activity and loss of body weight (expressed as percentage of body weight of the previous week) in KPC mice with various degrees of body weight loss (*n* = 10 animals). Data are mean ± standard error of the mean. Statistical analyses were performed using Kruskal–Wallis or unpaired one‐way ANOVA with Dunn's or Bonferroni *post hoc* tests, respectively (*B, C, E–I*), unpaired *t* test (*D, N*), and linear regression analysis (*J–L, O, P*). Tests were two sided. *P < 0.05, **P < 0.01, ***P < 0.001, and ****P < 0.0001. vs. MC38 cells (B, C) or tumour (E, F).

Cells and pieces of frozen tissues were homogenized in TRIzol (Life Technologies #15596018). RNA was then isolated by adding chloroform, precipitated with isopropanol, washed 2–3 times with 75% ethanol, and finally resuspended in RNase/DNase free water. After measurement of RNA concentration (Thermo Fisher #Nanodrop 2000), 1 μg of RNA was treated with DNase and reverse transcribed into cDNA (Qiagen #205313). Real‐time quantitative PCR was performed using Applied Biosystems QuantStudio 6 Flex Real‐Time PCR System (Applied Biosystems, #4485691). Gene expression was normalized to *Tbp* (cancer cell lines, tumours, GC muscles, hearts, livers, spleen, lymph nodes, and leucocytes) and *Hprt* (iWAT and eWAT) mRNA levels. For the different cancer cell lines (*Figure* 1B and Supporting Information, *Figures* S2) and tumours (*Figure* 1D), *Tbp* generated similar Ct values for a same amount of cDNA with a variability <0.9 Ct, allowing for a comparison of the 2^−ΔCt^ values. For tissues comparison (*Figures* 1E and 1F), Ct values of *Pla2g7* were directly compared for a same amount of cDNA as different control genes were used. We ensured similar loading of cDNA between animals by comparable Ct values of *Tbp* and *Hprt*. *Tbp* (Mm01277042_m1), *Crip1* (Mm01740674_g1), *Cdh13* (Mm00490584_m1), *Ezr* (Mm00447761_m1), *Spp1* (Mm00436767_m1), *S100a4* (Mm00803371_m1), *Gsn* (Mm00456679_m1), *Tgfbi* (Mm01337605_m1), *Sdpr* (Mm00507087_m1), *Lxn* (Mm00497417_m1), *Mgp* (Mm00485009_m1), *Arhgdib* (Mm00801450_m1), *Foxo3* (Mm01185722_m1), *Trim63* (Mm01185221_m1), *Fbxo32* (Mm00499523_m1), *Gabarapl1* (Mm00457880_m1), *Map 1lc3b* (Mm00782868_sH), and *Ctsl* (Mm00515597_m1) mRNA levels were determined using TaqMan Gene Expression Master Mix (Life Technologies #4369514). *Pla2g7* (F: 5′‐CACTGGCAAGACACATCTTC‐3′; R: 5′‐ATCAGATCTGTACAACCGAC‐3′), *Hprt* (F: 5′‐TGGCCATCTGCCTAGTAAAGC‐3′, R: 5′‐GGACGCAGCAACTGACATTTC‐3′), and *Saa1/2* (F: 5′‐GGAGTCTGGGCTGCTGAGAAAA‐3′; R: 5′‐TGTCTGTTGGCTTCCTGGTCAG‐3′) mRNA expression was assessed using PowerUp SYBR Green Master Mix (Life Technologies #A25776).

### Statistical analysis

Results from biological replicates were expressed as mean ± standard error of the mean. Statistical analysis was performed using GraphPad Prism 8.4. Normality was tested using D'Agostino–Pearson and Shapiro–Wilk normality tests. Statistical tests were two sided. Paired or unpaired Student's *t*‐tests and Mann–Whitney test were performed to compare two conditions. Paired or unpaired one‐way analysis of variance (ANOVA) or Kruskal–Wallis tests with Bonferroni and Dunn's *post hoc* tests, respectively, were applied to compare several groups. Paired two‐way ANOVA with Bonferroni *post hoc* tests were used to compare two variables. Linear regression analysis was performed to test association between two variables. Log‐rank (Mantel–Cox) test was used to compare time curves of cachexia development between groups. Finally, the receiver operating characteristic curve analysis, which measures the potential of a factor to discriminate between two populations based on its sensitivity and specificity, was performed using GraphPad Prism.

## Results

### High circulating levels of PLA2G7 are a hallmark of cancer cachexia in different mouse models with various tumour entities

In order to identify tumour‐borne factors involved in CCx, we took advantage of an innovative quantitative secretome analysis method combining click chemistry, pulsed stable isotope amino acid labelling, and mass spectrometry detection.^17^ This technology allowed us to identify proteins differentially secreted between the cachexia‐inducing colon carcinoma cell line C26 and the non‐cachexia‐inducing colon carcinoma cell line MC38 (*Figure* 1A and *Table* S1). Applying a threshold of a log_2_ fold change >2, most of the proteins enriched in C26 conditioned media (CM) belonged to cytoskeleton and/or were potentially involved in cell adhesion, migration, organization, and motility processes (CDH13, EZR, SPP1, S100A4, GSN, TGFBI, SDPR, MGP, and ARHGDIB), suggesting that they may be involved in cancer cell biology. A few other proteins were related to intracellular zinc trafficking and signalling (CRIP1 and ARHGDIB). Finally, some proteins were potentially related to inflammation (PLA2G7, SPP1, and LXN). To confirm an association between these proteins and the cachexia‐inducing properties of cancer cells, we then measured their expression levels in various murine cancer cells. The carcinoma C26 and LLC cells are well‐established models of CCx, inducing significant loss of body weight in tumour‐bearing mice, whereas MC38 and NC26 colon carcinoma cells do not affect body weight despite developing tumours of comparable size upon subcutaneous implantation (*Figures* S1A–S1N). Of note, data concerning tissues and tumour weights of control PBS, C26, and LLC‐injected mice shown in *Figures* S1M and S1N can be found in an original article previously published by our laboratory.^19^ These *in vivo* observations on skeletal muscle and adipose tissue weights were also reflected *in vitro*, with significant inductions of adipocyte lipolysis and myotube atrophy upon treatment with CM from C26 cells compared with CM from MC38 and NC26 cells or normal media (*Figures* S1O and S1P). Similarly, CM from pancreatic carcinoma 8025 cells have the potential to induce adipocyte lipolysis and myotube atrophy *in vitro* to a same extent as CM from C26 cells, whereas CM from pancreatic carcinoma Panc02 cells do not (*Figures* S1O and S1P). Interestingly, the phospholipase A2 group VII (PLA2G7; also known as lipoprotein‐associated phospholipase A2 or as PAF‐AH) was the only protein to show a clear differential expression profile between cachexia‐inducing cell lines (C26, LLC, and 8025) and non‐cachexia‐inducing cell lines (MC38, NC26, and Panc02) (*Figures* 1B and S2). High *Pla2g7* expression was associated with higher secretion, the latter assessed by measuring the specific PAF‐AH activity of PLA2G7 in CM (*Figure* 1C). *In vivo*, *Pla2g7* expression was also higher in C26 tumours than in MC38 tumours (*Figure* 1D). While *Pla2g7* expression in MC38 tumours was rather similar to the metabolic tissues involved in CCx, its expression in C26 tumours was markedly elevated (*Figures* 1E and 1F), suggesting that tumour could represent a significant source of circulating PLA2G7 in C26 tumour‐bearing mice.

Next, we determined whether circulating PLA2G7 levels were altered in different well‐established mouse models of CCx with various tumour entities. We compared cachectic C26 tumour‐bearing mice either to non‐cachectic MC38 and NC26 tumour‐bearing mice or to pre‐cachectic (i.e. before onset of body weight loss) C26 tumour‐bearing mice (*Figures* S1A–S1M and Morigny *et al*.^19^). Plasma PAF‐AH activity was greatly increased in cachectic C26 mice in comparison with non‐cachectic MC38, NC26, and PBS‐injected (vehicle control) mice (*Figures* 1G and 1H). Interestingly, pre‐cachectic C26 tumour‐bearing mice exhibited intermediate activity levels, between non‐cachectic PBS‐injected and cachectic C26 tumour‐bearing mice (*Figure* 1I), showing that circulating PLA2G7 progressively rises with cachexia development. We found close associations between circulating PAF‐AH activity and the extent of weight loss as well as white adipose tissue and skeletal muscle mass in all comparisons including non‐cachectic, pre‐cachectic, and cachectic mice (*Figures* 1J–1L and S3A–S3C). To assess further the biomarker potential of PLA2G7 in CCx, we performed a longitudinal prospective study in which we monitored circulating PLA2G7 levels in the course of cachexia development (*Figures* 1M, S1K, and S1L). Plasma PAF‐AH activity already discriminated non‐cachectic C26 tumour‐bearing mice from control PBS and NC26 tumour‐bearing mice only 12 days after cancer cell injection. Even more interestingly, circulating PLA2G7 levels were able to predict the time of CCx development in C26 tumour‐bearing mice independently of their tumour size (*Figures* S1K and S1L). We finally extended our observations to other cancer types by measuring circulating PAF‐AH activity in mice either subcutaneously injected with LLC cells or carrying genetic mutations of genes coding for the oncogene KRAS and tumour suppressor TRP53 in the pancreas, leading to spontaneous tumourigenesis (KPC mice). Similar to cachectic C26 mice, cachectic LLC tumour‐bearing mice had higher levels of plasma PAF‐AH activity than control PBS‐injected mice (*Figures* 1N and S1N and Morigny *et al*.^19^), and the circulating activity was also closely associated with loss of body weight, white adipose tissue, and skeletal muscle mass (*Figures* 1O and S3A–S3C). Furthermore, plasma PAF‐AH activity in KPC mice was also associated with body weight loss and tissue wasting (*Figures* 1P and S3A–S3C). Overall, these data show that increased circulating PLA2G7 levels are a hallmark of CCx in mice representing different tumour entities.

### *Pla2g7* knock‐down in C26 tumours slightly reduced circulating levels of PLA2G7 and did not affect cancer cachexia development

**Figure 2:**
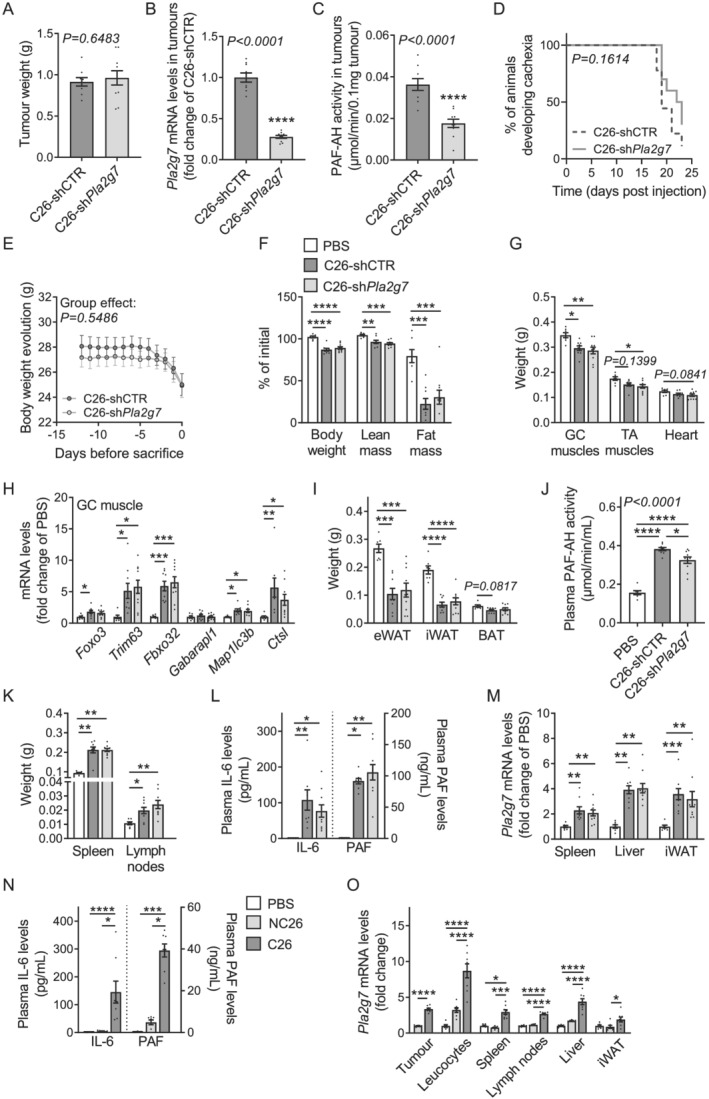
*Pla2g7* knock‐down in C26 tumours slightly reduced circulating levels of PLA2G7 and did not affect CCx development. *(A–M)* Mice were injected subcutaneously with PBS (control mice, white bars, *n* = 7 animals), control C26 cancer cells (C26‐shCTR, dark grey bars/lines, *n* = 9 animals), or C26 cancer cells stably knocked down for *Pla2g7* (C26‐sh*Pla2g7*, light grey bars/lines, *n* = 10 animals). *(A)* Tumour weights. *(B)* *Pla2g7* mRNA levels and *(C)* PAF‐AH activity in tumours. *(D)* Kaplan–Meier curve depicting the percentage of mice developing cachexia over time. *(E)* Kinetic of body weight loss during days prior sacrifice. *(F)* Loss of body weight, lean and fat mass (expressed as percentage of initial mass). *(G)* GC muscles, TA muscles, and heart weights. *(H)* mRNA levels of atrophy and autophagy markers in GC muscle. *(I)* Epididymal (eWAT), inguinal (iWAT), and brown (BAT) adipose tissues weights. *(J)* PAF‐AH activity in plasma. *(K)* Spleen and lymph nodes weights. *(L)* Plasma interleukin‐6 (IL‐6) and platelet‐activating factor (PAF) levels (*n* = 5 PBS animals, *n* = 8 C26‐shCTR animals, and *n* = 10 C26‐sh*Pla2g7* animals). *(M)* *Pla2g7* mRNA levels in spleen, liver, and iWAT. *(N)* Plasma IL‐6 and PAF levels and *(O)* *Pla2g7* mRNA levels in tumours, circulating leucocytes, and various tissues of PBS (white bars, *n* = 6 animals), non‐cachectic NC26 (light grey bars, *n* = 7 animals), and cachectic C26 (dark grey bars, *n* = 8 animals) tumour‐bearing mice. Data are mean ± standard error of the mean. Statistical analyses were performed using unpaired *t*‐test (*A–C*), unpaired one‐way ANOVA or Kruskal–Wallis with Bonferroni or Dunn's *post hoc* tests, respectively (*F–O*), paired two‐way ANOVA (*E*), and log‐rank (Mantel–Cox) test (*D*). Tests were two sided. *P < 0.05, **P < 0.01, ***P < 0.001, and ****P < 0.0001.

PLA2G7 is a calcium‐independent phospholipase A2 able to hydrolyze short‐chain and oxidized phospholipids as well as PAF. It can therefore assume both pro‐inflammatory and anti‐inflammatory effects and has been associated with numerous inflammatory diseases in humans.^11^, ^12^ To determine the relative contribution of PLA2G7 to tissue wasting in cancer, we generated C26 cells with a stable knock‐down for *Pla2g7* (C26‐sh*Pla2g7*), through the transfection of a plasmid expressing a shRNA against *Pla2g7* and subsequent selection of transfected cells. C26‐sh*Pla2g7* cells exhibited an 80% decrease in PLA2G7 expression and secretion and had a comparable growth as C26 cells transfected with a control shRNA (C26‐shCTR) (*Figures* S4A–S4C). *In vitro*, PLA2G7 depletion in C26 CM partially prevented myotube atrophy without affecting adipocyte lipolysis (*Figures* S4D and S4E). In addition to the potential direct effect of PLA2G7 on tissues, we also hypothesized that PLA2G7 may indirectly affect tissue wasting *in vivo* through the promotion of systemic inflammation. To address these hypotheses, we injected mice subcutaneously either with control C26 or C26‐sh*Pla2g7* cells. C26‐sh*Pla2g7* tumour‐bearing mice developed tumours of similar size as C26‐shCTR tumour‐bearing mice (*Figures* 2A and S4F) and displayed an approximately 75% and 50% reduction in *Pla2g7* expression and PAF‐AH activity in tumours, respectively (*Figures* 2B and 2C). Concerning CCx pathogenesis in this model, C26‐sh*Pla2g7* tumour‐bearing mice developed cachexia around the same time and to a similar extent as control C26 mice (*Figures* 2D–2F, S4G, and S4H). These mice had a comparable reduction in heart, GC, and TA muscles mass, confirmed by a similar increase in muscle expression of usual atrophy (*Foxo3*, *Trim63*, and *Fbxo32*) and autophagy (*Gabarapl1*, *Map1lc3b*, and *Ctsl*) markers involved in CCx (*Figures* 2G and 2H). They also showed no modification in adipose tissues mass (*Figures* 2F and 2I) and had comparable alterations of various plasma parameters (*Table* S2). Of note, food intake was unchanged between the two groups of tumour‐bearing mice (*Figure* S4I). Surprisingly, despite an important reduction of PAF‐AH activity in tumours (*Figure* 2C), C26‐sh*Pla2g7* mice only showed an around 20% decrease in plasma PAF‐AH activity levels (*Figure* 2J), suggesting that tumour may not be the only contributor to the circulating amounts of PLA2G7 in cachectic mice. As PLA2G7 is known to be secreted by monocytes/macrophages, mast cells, T cells, and other tissue‐resident cell types in different inflammatory diseases,^11^, ^12^, ^20^ the overall pro‐inflammatory status might have an important contribution to elevated PLA2G7 levels in CCx. This was supported by comparable enlargements of spleen and lymph nodes and by similar increases in circulating levels of the pro‐inflammatory factors IL‐6 and PAF in C26‐sh*Pla2g7* and C26‐shCTR mice (*Figures* 2K and 2L). Accordingly, the comparable inflammatory status of C26 mice was associated with a similar upregulation of *Pla2g7* expression in several tissues such as spleen, liver, and iWAT (*Figure* 2M). To support these observations further, we also measured circulating IL‐6 and PAF levels as well as *Pla2g7* expression in tumours, leucocytes, and a panel of tissues associated with inflammation including spleen, lymph nodes, liver, and iWAT, in PBS, non‐cachectic NC26, and cachectic C26 tumour‐bearing mice as characterized in *Figures* 1H and S1F–S1J. As expected, only C26 mice had a strong pro‐inflammatory phenotype and showed important increases in *Pla2g7* expression in all the tissues considered (*Figures* 2N and 2O). Altogether, these data suggest that tumour is not the only contributor to the circulating PLA2G7 levels in CCx and that the systemic pro‐inflammatory status may promote *Pla2g7* expression and secretion by various cell types from the host.

### Darapladib treatment was not sufficient to counteract cancer cachexia in C26 tumour‐bearing mice despite a strong inhibition of circulating PLA2G7 activity

**Figure 3:**
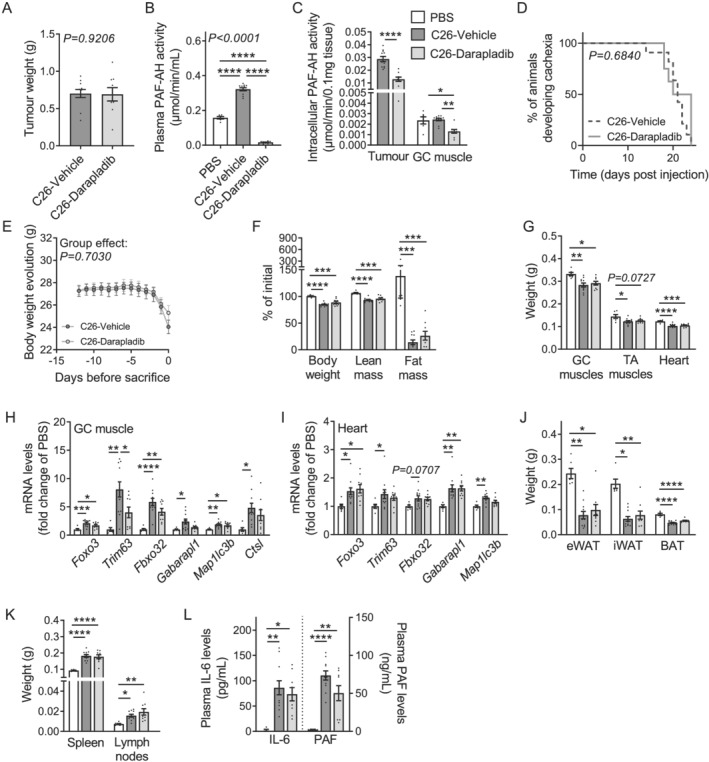
Darapladib treatment was not sufficient to counteract CCx in C26 tumour‐bearing mice despite a strong inhibition of PLA2G7 activity. *(A–L)* Mice were injected subcutaneously either with PBS (control mice) or C26 cancer cells and treated once daily either with vehicle (PBS mice, white bars, *n* = 6 animals; C26‐vehicle tumour‐bearing mice, dark grey bars/lines, *n* = 11 animals) or 50 mg/kg darapladib (C26‐darapladib tumour‐bearing mice, light grey bars/lines, *n* = 9 animals). *(A)* Tumour weights. *(B)* PAF‐AH activity in plasma, *(C)* tumours and GC muscles. *(D)* Kaplan–Meier curve depicting the percentage of mice developing cachexia over time. *(E)* Kinetic of body weight loss during days prior sacrifice. *(F)* Loss of body weight, and lean and fat mass (expressed as percentage of initial mass). *(G)* GC muscles, TA muscles, and heart weights. *(H)* mRNA levels of atrophy and autophagy markers in GC muscle and *(I)* heart. *(J)* Epididymal (eWAT), inguinal (iWAT), and brown (BAT) adipose tissues weights. *(K)* Spleen and lymph nodes weights. *(L)* Plasma interleukin‐6 (IL‐6) and platelet‐activating factor (PAF) levels (*n* = 5 PBS animals, *n* = 11 C26‐shCTR animals, and *n* = 9 C26‐sh*Pla2g7* animals). Data are mean ± standard error of the mean. Statistical analyses were performed using unpaired *t*‐test (*A, C*), unpaired one‐way ANOVA or Kruskal Wallis with Bonferroni or Dunn's *post hoc* tests, respectively (*B, C, F–L*), paired two‐way ANOVA (*E*), and log‐rank (Mantel–Cox) test (*D*). Tests were two sided. *P < 0.05, **P < 0.01, ***P < 0.001, and ****P < 0.0001.

As the rather mild reduction in circulating PLA2G7 activity upon genetic knock‐down in tumours did not allow for a clear conclusion about its potential functional impact on the pathogenesis of CCx, we next assessed a more global PLA2G7 inhibition paradigm. Darapladib and rilapladib are potent reversible inhibitors of PLA2G7 that have previously been tested in large cohorts of patients for treatment of various pathologies including cardiovascular diseases, diabetic retinopathy, and Alzheimer's disease.^13^, ^14^, ^15^, ^16^ Similar to *Pla2g7* knock‐down, inhibition of PLA2G7 in C26 CM using darapladib partly counteracted *in vitro* myotube atrophy but had no effect on adipocyte lipolysis (*Figures* S5A–S5C). *In vivo*, once tumours were palpable, C26 tumour‐bearing mice were treated once daily either with a vehicle solution or darapladib at a dose that efficiently inhibited circulating PLA2G7 activity over 24 h (*Figure* S5D,^21^, ^22^). Darapladib‐treated mice developed tumours of similar sizes as C26 mice treated with vehicle (*Figures* 3A and S5E) and had comparable food and water intakes (*Figures* S5F and S5G). The similar liver weights and mRNA levels of *Saa1/2* (encoding for the acute‐phase response protein *serum amyloid A*) (*Figures* S5H and S5I), as well as the circulating levels of AST and ALT (*Figures* S5J and S5K), suggested no acute liver toxicity in darapladib‐treated mice. After 2–3 weeks of treatment, darapladib‐treated mice still showed a 95% inhibition of circulating PLA2G7 activity 4 h after the last administration and a two times reduction in intra‐tissue activity (*Figures* 3B and 3C). Despite efficient inhibition, darapladib treatment did neither delay cachexia development nor decreased cachexia severity of C26 mice (*Figures* 3D–3F, S5L, and S5M). Darapladib‐treated mice were not protected against the loss of heart, GC, and TA muscles mass, as confirmed by unaffected expression of atrophy and autophagy markers in these tissues (*Figures* 3G–3I). These mice also showed a significant reduction in fat mass (*Figures* 3F and 3J) and displayed similar alterations of various plasma parameters such as blood glucose or cholesterol (*Table* S3). Finally, darapladib treatment did not improve the pro‐inflammatory status of C26 mice, as illustrated by the comparable enlargement of spleen and lymph nodes as well as the similar increase in circulating IL‐6 levels (*Figures* 3K and 3L). Of note, as reported in previous studies,^21^, ^22^ darapladib treatment had no effect on circulating PAF levels, questioning a central role of PLA2G7 on PAF function (*Figure* 3L). These data therefore do not support the use of PLA2G7 inhibitors for treatment of CCx, at least not as monotherapy.

### Circulating PLA2G7 levels are increased in weight‐losing cancer patients

**Figure 4:**
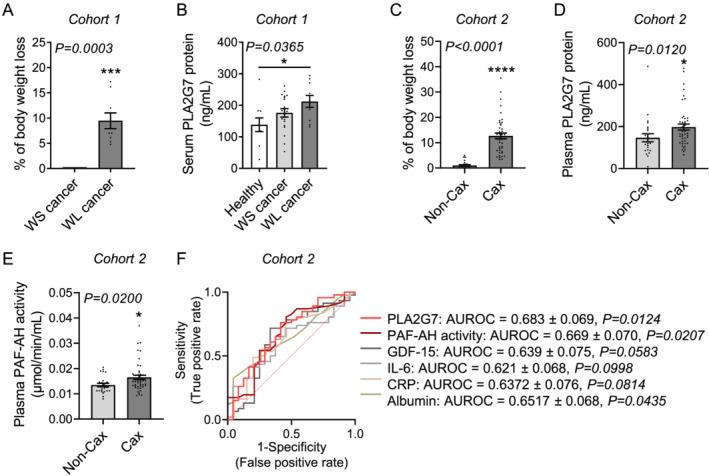
Circulating PLA2G7 levels are higher in weight‐losing cancer patients. *(A)* Percentage of body weight loss in the past 3 months and *(B)* PLA2G7 protein levels in serum of healthy individuals (white bar, *n* = 10 individuals) and weight‐stable (WS, light grey bars, *n* = 19 individuals) and weight‐losing (WL, dark grey bars, *n* = 10 individuals) patients with pancreatic or colorectal cancer. *(C)* Percentage of body weight loss, *(D)* plasma PLA2G7 protein, and (*E*) plasma PAF‐AH activity levels in non‐cachectic (Non‐Cax, light grey bars, *n* = 24 individuals) and cachectic (Cax, dark grey bars, *n* = 46 individuals) patients with pancreatic cancer. *(F)* Receiver operating characteristic (ROC) curve analysis, which measures the potential of circulating PLA2G7 protein, PAF‐AH activity, growth differentiation factor 15 (GDF‐15), interleukin‐6 (IL‐6), C‐reactive protein (CRP), or albumin levels to discriminate between cachectic and non‐cachectic cancer patients from Cohort 2 based on their sensitivity (true positive rate) and specificity (false positive rate: 1 − specificity). The area under the ROC curve (AUROC) and its associated *P* value illustrate the strength of a factor to distinguish between the two groups of patients. Data are mean ± standard error of the mean. Statistical analyses were performed using unpaired *t*‐test (*A*), Mann–Whitney test (*C–E*), and unpaired one‐way ANOVA with Bonferroni *post hoc* tests (*B*). ROC curve analysis (*F*) was performed using GraphPad Prism 8.4. Tests were two sided. *P < 0.05, ***P < 0.001, and ****P < 0.0001.

**Figure 5:**
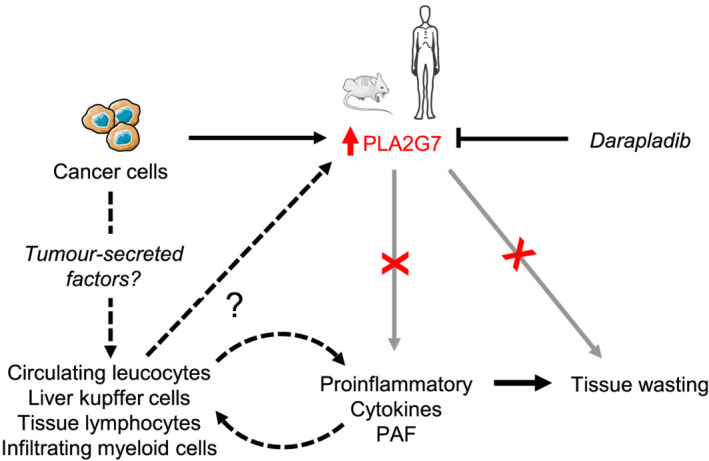
Graphical summary of the study. Circulating PLA2G7 levels are increased in CCx in both mice and humans. Despite a significant contribution, tumour is probably not the only responsible for the increase in circulating PLA2G7 levels in CCx. Circulating leucocytes and tissues such as liver, spleen, lymph nodes, or adipose tissue also show increased *Pla2g7* expression upon cachexia and may significantly contribute to its circulating levels. In tissues, liver Kupffer cells, lymphocytes, and infiltrating myeloid cells are considered to be the main cell types expressing *Pla2g7* (according to publicly available single cell RNA sequencing data on the Tabula Muris website, https://tabula‐muris.ds.czbiohub.org/). Tumour‐secreted factors as well as increased circulating levels of pro‐inflammatory cytokines and/or platelet‐activating factor (PAF) may promote PLA2G7 expression and secretion by cells from the host. Chronic treatment with the specific PLA2G7 inhibitor darapladib was not sufficient to improve inflammation and to counteract tissue wasting. Future studies should focus on the potential of PLA2G7 as early biomarker for the diagnosis of CCx.

To determine whether PLA2G7 is also a marker of CCx in humans, we measured its circulating levels in different cohorts of cancer patients with different tumour entities and degrees of body weight loss. In a first cohort of patients with pancreatic and colorectal cancer (*Table* 1), plasma PLA2G7 protein levels were significantly higher in patients who experienced weight loss within the past 3 months (i.e. weight‐losing) compared with healthy individuals (*Figures* 4A and 4B). Interestingly, cancer patients who did not suffer from body wasting (i.e. weight‐stable) exhibited an intermediary profile between healthy individuals and weight‐losing cancer patients. However, some limitations in this study arise from the classification of cancer patients and from the younger age of healthy individuals (see Materials and methods section for details). In a second larger cohort of pancreatic cancer patients classified according to the definition of cachexia by Fearon *et al*. (*Table* 2), both circulating PLA2G7 protein and activity levels were increased in cachectic compared with non‐cachectic cancer patients (*Figures* 4C–4E). Of note, PLA2G7 protein and activity levels were closely correlated (*Figure* S6A). When we stratified patients by gender, we observed a stronger association between CCx and circulating PLA2G7 levels in women than in men, although men also exhibit a trend to increased PLA2G7 protein levels in CCx (*Figures* S6B and S6C). We then performed a receiver operating characteristic curve analysis, which measures the ability of a factor to discriminate between two groups of patients by considering its sensitivity and specificity. Noteworthy, both PLA2G7 protein [area under the receiver operating characteristic curve (AUROC) = 0.683; *P* = 0.0124] and PAF‐AH activity (AUROC = 0.669; *P* = 0.0207) levels significantly distinguished cachectic and non‐cachectic patients and showed a better performance to classify patients than albumin (AUROC = 0.652; *P* = 0.0435) or the pro‐inflammatory C‐reactive protein (CRP, AUROC = 0.637; *P* = 0.0814), two markers usually used in clinical practice for diagnosis of CCx (*Figure* 4F). Of note, CRP and albumin are not used as stand‐alone markers for CCx diagnosis. We also compared the ability of PLA2G7 to discriminate between cachectic and non‐cachectic cancer patients to two other factors proposed as biomarkers for CCx diagnosis, IL‐6 and GDF‐15.^23^ PLA2G7 protein and PAF‐AH activity were also more efficient to stratify patients than IL‐6 (AUROC = 0.621; *P* = 0.0998) and GDF‐15 (AUROC = 0.639; *P* = 0.0583) (*Figures* 4F, S6D, and S6E). Future longitudinal prospective studies on larger cohorts of cancer patients, controlled for intervention strategies and co‐morbidities, will be necessary to confirm the predictive potential of PLA2G7 in combination with other established markers for CCx diagnosis. Altogether, these data show that despite no immediate pathogenic role, PLA2G7 is a consistent marker of CCx in both mice and humans (*Figure* 5).

## Discussion

Cancer cachexia is a multi‐organ syndrome notably characterized by the activation of inflammatory and catabolic pathways and the inhibition of anabolic pathways, thereby reducing patient tolerance and responsiveness to anticancer therapies.^1^ Despite efforts in the past years to identify molecular mechanisms involved in CCx, diagnosis and therapeutic tools are still lacking for patient care. Here, we identified the phospholipase PLA2G7 as a consistent marker of CCx in both well‐established mouse models of CCx and cachectic cancer patients with various tumour entities. In the circulation, PLA2G7 binds to lipoproteins and has the particularity of harbouring a calcium‐independent activity.^11^, ^12^ PLA2G7 hydrolyzes the pro‐inflammatory factor PAF, leading to its inactivation into lyso‐PAF, as well as phospholipids carrying *sn*‐2 short‐chain or oxidized long‐chain fatty acids, releasing fragments of phospholipids such as lysophosphatidylcholines (LPCs). Depending on its substrate, PLA2G7 would therefore exert anti‐inflammatory or pro‐inflammatory effects, through PAF degradation and LPCs/oxidized fatty acids generation, respectively. Indeed, LPCs have been associated with recruitment of monocytes and production of pro‐inflammatory cytokines *in vitro*.^24^, ^25^, ^26^ Therefore, PLA2G7 has been extensively studied in many inflammatory diseases, and the PLA2G7 inhibitors darapladib or rilapladib have already been tested in Phase II and Phase III clinical trials for treatment of Alzheimer's disease,^14^ diabetic retinopathy,^16^ and atherosclerosis.^13^, ^15^ In this study, darapladib treatment, despite efficient inhibition of circulating PLA2G7 activity, was not sufficient as a unique therapy to reduce inflammation and tissue wasting in C26 tumour‐bearing mice. Accordingly, we recently reported reduced plasma levels of various LPC species in different mouse models of CCx as well as cachectic cancer patients,^19^ suggesting that PLA2G7 is not crucial for LPC turnover in CCx. This is in agreement with recent reviews that questioned the pro‐inflammatory effects of PLA2G7 through LPC production.^20^, ^27^, ^28^ Considering the latter, it may explain in part why darapladib treatment has been inefficient in preventing cardiovascular death, myocardial infarction, stroke, or major coronary events in patients with coronary heart diseases.^13^, ^15^ It is still possible that PLA2G7 enzymatic activity might require additional pro‐cachectic pathways to functionally impact cachectic phenotypes. Furthermore, another potential effect of PLA2G7 in CCx relates to cachexia‐associated thrombosis. Cancer patients have an increased risk of developing venous thrombosis,^29^, ^30^ which represents a major cause of cancer‐related morbidity and mortality.^31^, ^32^, ^33^ Increased circulating levels of coagulation factors and platelets have been reported in cachectic C26 tumour‐bearing mice, and hypercoagulability in this model is partially IL‐6 dependent.^34^ Whether PLA2G7 might prevent or worsen cachexia‐associated thrombosis through PAF hydrolysis or generation of pro‐inflammatory lipid species, respectively, is unknown. However, the absence of an effect of darapladib on PAF levels in this study and previous reports^21^, ^22^ as well as the growing debate concerning the pro‐inflammatory function of PLA2G7 through LPC production^20^, ^27^, ^28^ challenge the hypothesis that PLA2G7 is involved in cachexia‐associated thrombosis.

An important issue regarding PLA2G7 is whether its increase is a cause or a consequence of inflammation in several pathologies.^20^, ^27^, ^28^ Here, we show that, despite a significant contribution, tumour is probably not the only source of circulating PLA2G7 in cachectic tumour‐bearing mice. We believe that immune cells and other tissue‐resident cell types from the host also play an important part to the circulating levels of PLA2G7 in CCx as they do in other inflammatory diseases.^11^, ^12^, ^35^ It has been shown that PLA2G7 expression and secretion is induced during differentiation of monocyte into macrophage and during polarization into M1 macrophages.^11^, ^12^, ^20^ Several mediators could be involved in the regulation of *Pla2g7* expression *in vivo*. One could hypothesize that high PLA2G7 levels are a compensatory mechanism to higher PAF levels. Regulation by cytokines *in vitro* is controversial and may depend on the differentiation stage of the cells. Furthermore, other tumour‐secreted factors, SAA, or oxidized LDL levels could also be involved in elevated *Pla2g7* expression in CCx. Of note, we previously reported higher circulating LDL levels in cachectic C26 vs. non‐cachectic NC26 tumour‐bearing mice.^19^ Here, we measured very high circulating levels of IL‐6 (as previously reported by Reddel *et al*.^34^) and PAF in cachectic C26 mice in comparison with non‐cachectic NC26 mice. This systemic pro‐inflammatory status mirrored stronger expressions of *Pla2g7* in leucocytes and different tissues from cachectic animals, suggesting that increased PLA2G7 levels in CCx may be the consequence of inflammation. As for some studies published on PLA2G7 and darapladib in the context of cardiovascular diseases,^20^, ^27^, ^28^ the current study does not highlight any crucial effect of PLA2G7 on CCx pathogenesis. Our data support previous findings in other diseases, suggesting that the increase in circulating PLA2G7 levels in CCx is the consequence of increased PAF and inflammatory cytokines levels and may represent a protective mechanism rather than a risk factor.

In addition to new therapeutic targets, identification of novel biomarkers of CCx is a prerequisite for an early detection of the disease and the success of a therapy.^2^, ^23^ Indeed, some therapeutic approaches showed encouraging significant results only in pre‐cachectic cancer patients with less advanced disease stage and malnutrition.^36^ Circulating PLA2G7 levels have already been proposed as biomarker for cardiovascular diseases and would be predictive of adverse cardiovascular events and ischaemic strokes in patients with stable coronary diseases^37^, ^38^, ^39^ and healthy individuals,^40^, ^41^ respectively. PLA2G7 has also been recently proposed as biomarker for COVID‐19‐related and pneumonia‐related cardiovascular complications.^35^ High PLA2G7 expression in tumours has been associated with aggressive types of prostate and breast cancers and with a poor survival rate of cancer patients.^42^, ^43^ We show in this study that PLA2G7 is also a hallmark of cachexia‐inducing tumours and that circulating PLA2G7 protein and activity levels are elevated in different mouse models of CCx as well as in cachectic cancer patients with pancreatic and colorectal cancer, supporting its potential as biomarker for different cancer types. Furthermore, circulating PLA2G7 levels progressively rise during cachexia development and are already increased in pre‐cachectic tumour‐bearing animals, suggesting that PLA2G7 could be an early marker of the disease (i.e. before onset of body weight loss). In comparison with cancer‐related biomarkers routinely used in clinic to discriminate between healthy and cancer patients, such as CA19‐9 for diagnosis of pancreatic cancer showing AUROC values comprised between 0.872 and 0.947,^44^ PLA2G7 was less efficient to discriminate between cachectic and non‐cachectic pancreatic cancer patients in the present study. It has to be kept into consideration that cachexia is a complex multifactorial syndrome that is still incompletely understood. It can evolve from pre‐cachexia to refractory cachexia, however, along diverse trajectories, and not every patient will progress to the most advanced stage of the disease. This may in part explain why, in contrast to different cancers, there is still no validated biomarker for CCx.^23^ Interestingly, PLA2G7 protein and activity levels were more efficient to discriminate between cachectic and non‐cachectic cancer patients than IL‐6 and GDF‐15, two other factors proposed as biomarkers for CCx diagnosis.^23^ These data suggest that PLA2G7 may be a potential biomarker for diagnosis of cachexia. However, its predictive value, as for other markers, may not be sufficient as stand‐alone marker to be able to diagnose patients susceptible to cachexia. Nevertheless, its predictive value, in combination with other markers such as albumin, CRP, GDF‐15, or IL‐6, for instance, may represent an efficient signature for diagnosis of cachexia. Future longitudinal prospective studies involving larger cohorts of patients with different types of cancers and controlled for intervention strategies and co‐morbidities are required to evaluate its potential as biomarker for CCx.

Overall, our work highlights PLA2G7 as a signature of cachexia‐inducing cancer cell lines. High circulating PLA2G7 levels are a consistent marker of CCx in both mice and patients carrying diverse tumour entities. Inhibitory treatment using the well‐studied inhibitor darapladib does not appear as a promising strategy for the care of cachectic cancer patients, at least not as a single therapy. The present study paves the way to further assessment of PLA2G7 as potential biomarker for early detection of CCx.

## Conflict of interest

None declared.

## Funding

P.M., J.M., M.B.D., and S.H. are supported by the German Research Foundation (Deutsche Forschungsgemeinschaft, SFB1321 (Project ID 329628492) and SFB824/3 2017). S.H. is supported by the Else Kröner‐Fresenius‐Stiftung (2020 EKSE.23). D.K. is supported by an Erwin Schrödinger Fellowship from the Austrian Science Fund (FWF, J4224‐B34). J.Z., M.R., and S.H. are supported by the Helmholtz Alliance ‘Aging and Metabolic Programming’ (AMPro). M.R. is supported by the DACH Gesellschaft Prävention von Herz‐Kreislauf‐Erkrankungen and EFSD/Boehringer Ingelheim European Research Programme on ‘Multi‐Systems Challenges in Diabetes’. S.F.S. was supported by a fellowship from the Novo Nordisk Fonden (NNFOC150019050). O.P. is supported by a Clinical Leave Stipend from the German Center for Infection Research [Deutsches Zentrum für Infektionsforschung (DZIF), Grant TI07.001]. H.K. was supported by a scholarship from doctoral program in Translational Medicine, Faculty of Medicine, Munich University of Technology (TUM School of Medicine).

## Supporting information


**Table S1.** Proteins differentially secreted in C26 versus MC38 conditioned media with a Log2 value of fold change (FC) > 2 and an adjusted P value > 0.05. *n* = 3 independent biological replicates per group.
**Table S2.** Plasma parameters of mice injected either with PBS (control mice, *n* = 4 animals), control C26 cancer cells (C26‐shCTR, *n* = 8 animals) or C26 cancer cells knocked down for Pla2g7 (C26‐shPla2g7, *n* = 8 animals). Data are mean ± standard error of the mean. Statistical analyses were performed using unpaired one‐way ANOVA with Bonferroni post‐hoc tests. Tests were two‐sided. * versus PBS. *p<0.05, **p<0.01, ***p<0.001.
**Table S3.** Plasma parameters of mice injected either with PBS (control mice) or C26 cancer cells, and treated once daily either with vehicle (PBS mice *n* = 4 animals, C26‐Vehicle tumour‐bearing mice *n* = 8 animals) or 50mg/kg darapladib (C26‐darapladib tumour‐bearing mice *n* = 8 animals). Data are mean ± standard error of the mean. Statistical analyses were performed using unpaired one‐way ANOVA with Bonferroni post‐hoc tests. Tests were two‐sided. * versus PBS. *p<0.05, **p<0.01, ***p<0.001, ****p<0.0001. NS = non‐significant.

**Figure S1.** (A‐E) Body weight loss (expressed as percentage of initial body weight) (A), epididymal (eWAT) (B) and inguinal (iWAT) (C) adipose tissues weights, GC muscles (D), and tumours (E) weights of PBS (white bars, *n* = 16 animals) non‐cachectic MC38 (light grey bars, *n* = 8 animals) and cachectic C26 (dark grey bars, *n* = 8 animals) tumour‐bearing mice. (F‐J) Body weight loss (expressed as percentage of initial body weight) (F), epididymal (eWAT) (G) and inguinal (iWAT) (H) adipose tissues weights, GC muscles (I), and tumours (J) weights of PBS (white bars, *n* = 6 animals) non‐cachectic NC26 (light grey bars, *n* = 7 animals) and cachectic C26 (dark grey bars, *n* = 8 animals) tumour‐bearing mice. (K‐L) Longitudinal prospective study showing the evolution of body weight (expressed as percentage of initial body weight) (K) and tumour volume (L) in PBS (light grey lines, *n* = 6 animals), non‐cachectic NC26 (dark grey lines, *n* = 7 animals) and cachectic C26 (red lines, *n* = 8 animals) tumour‐bearing mice throughout cachexia development (**mice presented in Figures S1F‐J**). C26 mice were divided into 3 groups based on their time course of cachexia development including an early (days 17‐18, dark red lines), late (days 20‐21, bright red lines) and very late (day 22, light red lines) cachexia development. The graphs show individual mice data. (M‐N) Body weight loss (expressed as percentage of initial body weight) of (M) PBS (white bar, *n* = 9 animals), pre‐cachectic (C26‐precax, light grey bar, *n* = 11 animals) and cachectic (C26‐cax, dark grey bar, *n* = 9 animals) C26 tumour‐bearing mice; and (N) PBS (white bar, *n* = 5 animals) and LLC tumour‐bearing mice (dark grey bar, *n* = 6 animals). (O‐P) Glycerol released in media of 3T3‐L1 adipocytes (*n* = 3‐6 biological replicates per group) (O) and diameters of C2C12 myotubes (*n* = 4‐9 biological replicates per group) (P) treated for 48 hours with normal media (control, CTR, white bars) or conditioned‐media from various cancer cell lines (light grey bars: no cachexia‐inducing properties; dark grey bars: cachexia‐inducing properties). Data are mean ± standard error of the mean. Statistical analyses were performed using one‐way ANOVA or Kruskal‐Wallis with Bonferroni or Dunn’s post‐hoc tests (A‐D, F‐I, M, O‐P) and unpaired t test (E, J, N) respectively. Tests were two‐sided. *p<0.05, **p<0.01, ***p<0.001, ****p<0.0001. * *versus* CTR (O‐P).
**Figure S2.** (A‐K) mRNA levels of (A) *Crip1*, (B), (B) *Cdh13*, (C) *Ezr*, (D) *Spp1*, (E) *S100a4*, (F) *Gsn*, (G) *Tgfbi*, (H) *Sdpr*, (I) *Lxn*, (J) *Mgp* and (K) *Arhgdib* in various cancer cell lines with different cachexia‐inducing properties (*n* = 3‐5 independent biological replicates per group; light grey bars: no cachexia‐inducing properties; dark grey bars: cachexia‐inducing properties). MC38/NC26/C26 are murine colon carcinoma cell lines; Panc02/8025 are murine pancreatic carcinoma cell lines. Data are mean ± standard error of the mean. Statistical analysis was performed using Kruskal Wallis with Dunn’s post‐hoc tests. Tests were two‐sided. *p<0.05, **p<0.01, ***p<0.001, ****p<0.0001.
**Figure S3.** (A‐C) Linear regression analyses comparing plasma PAF‐AH activity and loss of epididymal adipose tissue (eWAT) (A), inguinal adipose tissue (iWAT) (B) and GC muscles mass (C) in (from top to bottom): PBS (white dots, *n* = 10 animals), non‐cachectic MC38 (light grey dots, *n* = 5 animals) and cachectic C26 (dark grey dots, *n* = 5 animals) tumour‐bearing mice; PBS (white dots, *n* = 6 animals), non‐cachectic NC26 (light grey dots, *n* = 7 animals) and cachectic C26 (dark grey dots, *n* = 8 animals) tumour‐bearing mice; PBS (white dots, *n* = 9 animals), pre‐cachectic (C26‐precax, light grey dots, *n* = 11 animals) and cachectic (C26‐cax, dark grey dots, *n* = 9 animals) C26 tumour‐bearing mice; PBS (white dots, *n* = 5 animals) and LLC tumour‐bearing mice (dark grey dots, *n* = 6 animals); and KPC mice with various degrees of body weight loss (*n* = 10 animals). Of note, data from MC38 and C26 tumour‐bearing mice are from another cohort than the one presented in **Figure S1A‐E** but which shared the same properties (in terms of loss of body weight, fat and muscle mass for a similar tumour size). Statistical analyses were performed using linear regression analysis.
**Figure S4.** (A‐C) *Pla2g7* mRNA levels (*n* = 4 biological replicates per group) (A), PAF‐AH activity in conditioned media (*n* = 6 biological replicates per group) (B), and growth rate (*n* = 7 biological replicates per group) (C) of control C26 cancer cells (C26‐shCTR, dark grey bars) and C26 cancer cells stably knocked down for *Pla2g7* (C26‐sh*Pla2g7*, light grey bars). (D‐E) Diameters of C2C12 myotubes (*n* = 9 biological replicates per group) (D) and glycerol released in media of 3T3‐L1 adipocytes (*n* = 7 biological replicates per group) (E) treated for 48 hours with normal media (control, CTR, white bars) or conditioned media from C26‐shCTR (dark grey bars) and C26‐sh*Pla2g7* (light grey bars) cancer cells. (F‐I) Mice were injected either with PBS (control mice, white bars, *n* = 7 animals), control C26 cancer cells (C26‐shCTR, dark grey bars/lines, *n* = 9 animals) or C26 cancer cells stably knocked down for *Pla2g7* (C26‐sh*Pla2g7*, light grey bars/lines, *n* = 10 animals). (F) Tumour growth curve. (G) Initial body weight, lean and fat mass (i.e. at the time of cancer cells injection). (H) Reasons dictating the endpoint at which mice were sacrificed. 8 C26‐shCTR mice and 7 C26‐sh*Pla2g7* mice developed cachexia. 1 mouse per group was sacrificed because of tumour size reaching humane endpoint before developing cachexia (‐2.1% of body weight loss for the C26‐shCTR mouse and ‐2.8% for the C26‐sh*Pla2g7* mouse). The two last C26‐sh*Pla2g7* mice were sacrificed when the last mouse from the C26‐shCTR group developed cachexia. This refers to “end of experiment”. (I) Food intake over time post cancer cells injection. Data are mean ± standard error of the mean. Statistical analyses were performed using unpaired (A‐B) or paired (C) *t* tests, paired (D‐E) or unpaired (G) one‐way ANOVA with Bonferroni post‐hoc tests, and paired two‐way ANOVA (F, I). Tests were two‐sided. *p<0.05, **p<0.01, ***p<0.001, ****p<0.0001.
**Figure S5.** (A) PAF‐AH activity in C26 conditioned media supplemented with vehicle or 1μM darapladib (*n* = 2 biological replicates per group). (B‐C) Diameters of C2C12 myotubes (*n* = 8 biological replicates per group) (B) and (C) glycerol released in media of 3T3L1 adipocytes (*n* = 8 biological replicates per group) treated for 48 hours with normal media (control, white bars) or C26 conditioned media (grey bars) supplemented with vehicle or 1μM darapladib. (D) Plasma PAF‐AH activity over 24 hours in mice treated either with vehicle (*n* = 1 animal) or a single dose of 50mg/kg darapladib (*n* = 2 animals). (E‐M) Mice were injected either with PBS (control mice) or C26 cancer cells, and treated once daily either with vehicle (PBS mice, white bars, *n* = 6 animals; C26‐Vehicle tumour‐bearing mice, dark grey bars/lines, *n* = 11 animals) or 50mg/kg darapladib (C26‐Darapladib tumour‐bearing mice, light grey bars/lines, *n* = 9 animals). (E) Tumour growth curve. (F‐G) Food (F) and water (G) intakes over time post cancer cell injection. (H‐I) Liver weights (H) and liver *Saa1/2* mRNA levels (I). (J‐K) Plasma AST (J) and ALT (K) levels. (L) Initial body weight, lean and fat mass (i.e. at the time of cancer cells injection). (M) Reasons dictating the endpoint at which mice were sacrificed. 10 C26‐Vehicle mice and 5 C26‐Darapladib mice developed cachexia. 1 mouse per group was sacrificed because of tumour size reaching humane endpoint but already started to lose weight (‐8.5% body weight loss for the C26‐Vehicle mouse and ‐8% for the C26‐Darapladib mouse). 3 mice from the darapladib group were sacrificed because of tumour ulceration but already started to lose weight (‐4,3% ± 2% body weight loss). Data are mean ± standard error of the mean. Statistical analyses were performed using paired one‐way ANOVA with Bonferroni post‐hoc tests (B‐C), paired two‐way ANOVA (E‐G), unpaired one‐way ANOVA or Kruskal Wallis with Bonferroni or Dunn’s (H‐L) post‐hoc tests respectively. Tests were two‐sided. *p<0.05, **p<0.01, ***p<0.001, ****p<0.0001.
**Figure S6.** (A) Linear regression analysis comparing plasma PLA2G7 protein and PAF‐AH activity levels in non‐cachectic (Non‐cax, light grey dots, *n* = 24 individuals) and cachectic (Cax, dark grey dots, *n* = 46 individuals) patients with pancreatic cancer (cohort 2). (B‐C) Plasma PLA2G7 protein (B) and PAF‐AH activity (C) levels in non‐cachectic (Non‐cax, light grey bars, *n* = 24 individuals) and cachectic (Cax, dark grey bars, *n* = 46 individuals) patients classified by gender (cohort 2). (D‐E) Circulating levels of growth differentiation factor 15 (GDF‐15) (D) and interleukin 6 (IL‐6) in non‐cachectic (Non‐cax, light grey dots, *n* = 24 individuals) and cachectic (Cax, dark grey dots, *n* = 46 individuals) patients (cohort 2). Data are mean ± standard error of the mean. Statistical analyses were performed using linear regression (A) and Mann‐Whitney test (B‐E). Tests were two‐sided. *p<0.05.

Supplementary Material Legends
