# Supplementary material for: Association of circulating PLA2G7 levels with cancer cachexia and assessment of darapladib as a therapy
Source: J Cachexia Sarcopenia Muscle. 2021 Aug 23;12(5):1333–51. doi: 10.1002/jcsm.12758 (PMC8517355; doi:10.1002/jcsm.12758)
Supplement: Supplementary file 1 — Table S1. Proteins differentially secreted in C26 versus MC38 conditioned media with a Log2 value of fold change (FC) > 2 and an adjusted P value > 0.05. n = 3 independent biological replicates per group. Table S2. Plasma parameters of mice injected either with PBS (control mice, n = 4 animals), control C26 cancer cells (C26‐shCTR, n = 8 animals) or C26 cancer cells knocked down for Pla2g7 (C26‐shPla2g7, n = 8 animals). Data are mean ± standard error of the mean. Statistical analyses were performed using unpaired one‐way ANOVA with Bonferroni post‐hoc tests. Tests were two‐sided. * versus PBS. *p<0.05, **p<0.01, ***p<0.001. Table S3. Plasma parameters of mice injected either with PBS (control mice) or C26 cancer cells, and treated once daily either with vehicle (PBS mice n = 4 animals, C26‐Vehicle tumour‐bearing mice n = 8 animals) or 50mg/kg darapladib (C26‐darapladib tumour‐bearing mice n = 8 animals). Data are mean ± standard error of the mean. Statistical analyses were performed using unpaired one‐way ANOVA with Bonferroni post‐hoc tests. Tests were two‐sided. * versus PBS. *p<0.05, **p<0.01, ***p<0.001, ****p<0.0001. NS = non‐significant. [file JCSM-12-1333-s002.docx]

| Protein | Log2 FC (C26/MC38) | *P* value | Reported function (Uniprot / PubMed Gene) |
| --- | --- | --- | --- |
| CRIP1  *Cystein-rich protein 1* | 3.28 | 0.005 | Protein that may function as an intracellular zinc transport protein. |
| PLA2G7  *Platelet-activating factor acetylhydrolase* | 2.71 | 0.003 | Lipoprotein-associated calcium-independent phospholipase A2 involved in short-chain or oxidized phospholipid catabolism during inflammatory and oxidative stress response. PLA2G7 also hydrolyzes and inactivates platelet-activating factor, a potent proinflammatory signaling lipid that acts on various innate immune cells. |
| CDH13  *Cadherin-13* | 2.71 | 0.013 | Protein that belongs to the cadherins family, calcium-dependent cell adhesion proteins. Cadherins preferentially interact with themselves in a homophilic manner in connecting cells. They may act as negative regulator of neural cell growth. |
| EZR  *Erzin* | 2.61 | 0.001 | Protein probably involved in connections of major cytoskeletal structures to the plasma membrane. In epithelial cells, it is required for the formation of microvilli and membrane ruffles on the apical pole. This protein plays a key role in cell surface structure, adhesion, migration and organization. It has been implicated in multiple human cancers. |
| SPP1  *Osteopontin* | 2.58 | 0.007 | Protein that binds tightly to hydroxyapatite. It appears to form an integral part of the mineralized matrix. It is probably important for cell-matrix interaction. It also acts as a cytokine, enhancing production of interferon-γ and interleukin-12 and reducing production if interleukin-10. It plays a central part in type I immunity. |
| S100A4  *S100 calcium-binding protein A4* | 2.51 | 0.001 | S100 proteins are localized in the cytoplasm and/or nucelus of a wide-range of cells and are involved in the regulation of a number of cellular processes such as cycle progression and differentiation. It may function in motility, invasion and tubulin polymerization. |
| GSN  *Gelsolin* | 2.37 | 0.005 | Calcium-regulated-actin-modulating protein that binds to the plus ends of actin monomers or filaments, preventing monomer exchange. It can promote the assembly of monomers into filaments, as well as server filaments already formed. |
| TGFBI  *Transforming growth factor-beta-induced protein ig-h3* | 2.35 | 0.002 | Protein that binds to type I, II and IV collagens. The protein plays a role in cell-collagen interactions. It is induced by transforming growth factor-β and acts to inhibit cell adhesion. |
| SDPR  *Serum deprivation-response protein*  also known as C*avin-2* | 2.3 | 0.001 | Calcium-independent phospholipid-binding protein whose expression increases in serum-starved cells. Protein that plays an important role in caveolar biogenesis and morphology in a tissue-specific manner (i.e involved in caveolae formation in lung and fat endothelia but not in heart endothelia). Removal of this protein causes caveolae loss and its over-expression results in caveolae deformation and membrane tubulation. |
| LXN  *Latexin* | 2.27 | 0.013 | Hardly reversible, non-competitive and potent inhibitor of carboxypeptidases A1, A2 and A4. It may play a role in inflammation as it downregulates the population size of hematopoietic stem cells. |
| MGP  *Matrix Gla protein* | 2.03 | 0.005 | Protein that associates with the organic matrix of bone and cartilage. It is secreted by chondrocytes and vascular smooth muscle cells and functions as a physiological inhibitor of ectopic tissue calcification. |
| ARHGDIB  *Rho GDP-dissociation inhibitor 2* | 2.02 | 0.006 | Protein that regulates the GDP/GTP exchange reaction of Rho proteins by inhibiting the dissociation of GDP from them and the subsequent binding to GTP. It regulates the reorganization of the actin cytoskeleton mediated by Rho family members. |

**Table S1.** Proteins differentially secreted in C26 *versus* MC38 conditioned media with a Log2 value of fold change (FC) > 2 and an adjusted *P* value > 0.05. n=3 independent biological replicates per group.

|  | PBS | C26-shCTR | C26-sh*Pla2g7* |
| --- | --- | --- | --- |
| n | 4 | 8 | 8 |
| Total proteins (g/dL) | 41.9 (± 3.5) | 53.0 (± 1.4) ** | 52.7 (± 1.8) ** |
| Total cholesterol (mg/dL) | 99.8 (± 5.9) | 150.0 (± 7.1) ** | 147.0 (± 9.5) ** |
| HDL cholesterol (mg/dL) | 72.0 (± 5.4) | 95.3 (± 4.6) * | 97.4 (± 6.0) * |
| LDL cholesterol (mg/dL) | 11.1 (± 0.9) | 40.1 (± 3.2) *** | 38.6 (± 4.0) *** |
| Glucose (mg/dL) | 312.0 (± 24.6) | 143.8 (± 20.6) *** | 151.3 (± 20.1) *** |

**Table S2.** Plasma parameters of mice injected either with PBS (control mice, n=4 animals), control C26 cancer cells (C26-shCTR, n=8 animals) or C26 cancer cells knocked down for *Pla2g7* (C26-sh*Pla2g7*, n=8 animals). Data are mean ± standard error of the mean. Statistical analyses were performed using unpaired one-way ANOVA with Bonferroni post-hoc tests. Tests were two-sided. * *versus* PBS. *p<0.05, **p<0.01, ***p<0.001.

|  | PBS | C26-Vehicle | C26-Darapladib |
| --- | --- | --- | --- |
| n | 4 | 8 | 8 |
| Total proteins (g/dL) | 42.2 (± 1.4) | 54.4 (± 1.6) ** | 50.1 (± 1.9) * |
| Total cholesterol (mg/dL) | 126.3 (± 3.7) | 158.9 (± 7.5) * | 143.4 (± 6.9) NS |
| HDL cholesterol (mg/dL) | 87.1 (± 3.1) | 101.8 (± 5.1) NS | 92.8 (± 4.2) NS |
| LDL cholesterol (mg/dL) | 9.5 (± 0.8) | 30.1 (± 2.4) **** | 23.3 (± 2.1) ** |
| Glucose (mg/dL) | 291.5 (± 21.0) | 205.9 (± 9.2) *** | 217.1 (± 9.9) ** |

**Table S3.** Plasma parameters of mice injected either with PBS (control mice) or C26 cancer cells, and treated once daily either with vehicle (PBS mice n=4 animals, C26-Vehicle tumour-bearing mice n=8 animals) or 50mg/kg darapladib (C26-darapladib tumour-bearing mice n=8 animals). Data are mean ± standard error of the mean. Statistical analyses were performed using unpaired one-way ANOVA with Bonferroni post-hoc tests. Tests were two-sided. * *versus* PBS. *p<0.05, **p<0.01, ***p<0.001, ****p<0.0001. NS = non-significant.
