# Supplementary figures and images for: Association of circulating PLA2G7 levels with cancer cachexia and assessment of darapladib as a therapy
Source: J Cachexia Sarcopenia Muscle. 2021 Aug 23;12(5):1333–51. doi: 10.1002/jcsm.12758 (PMC8517355; doi:10.1002/jcsm.12758)

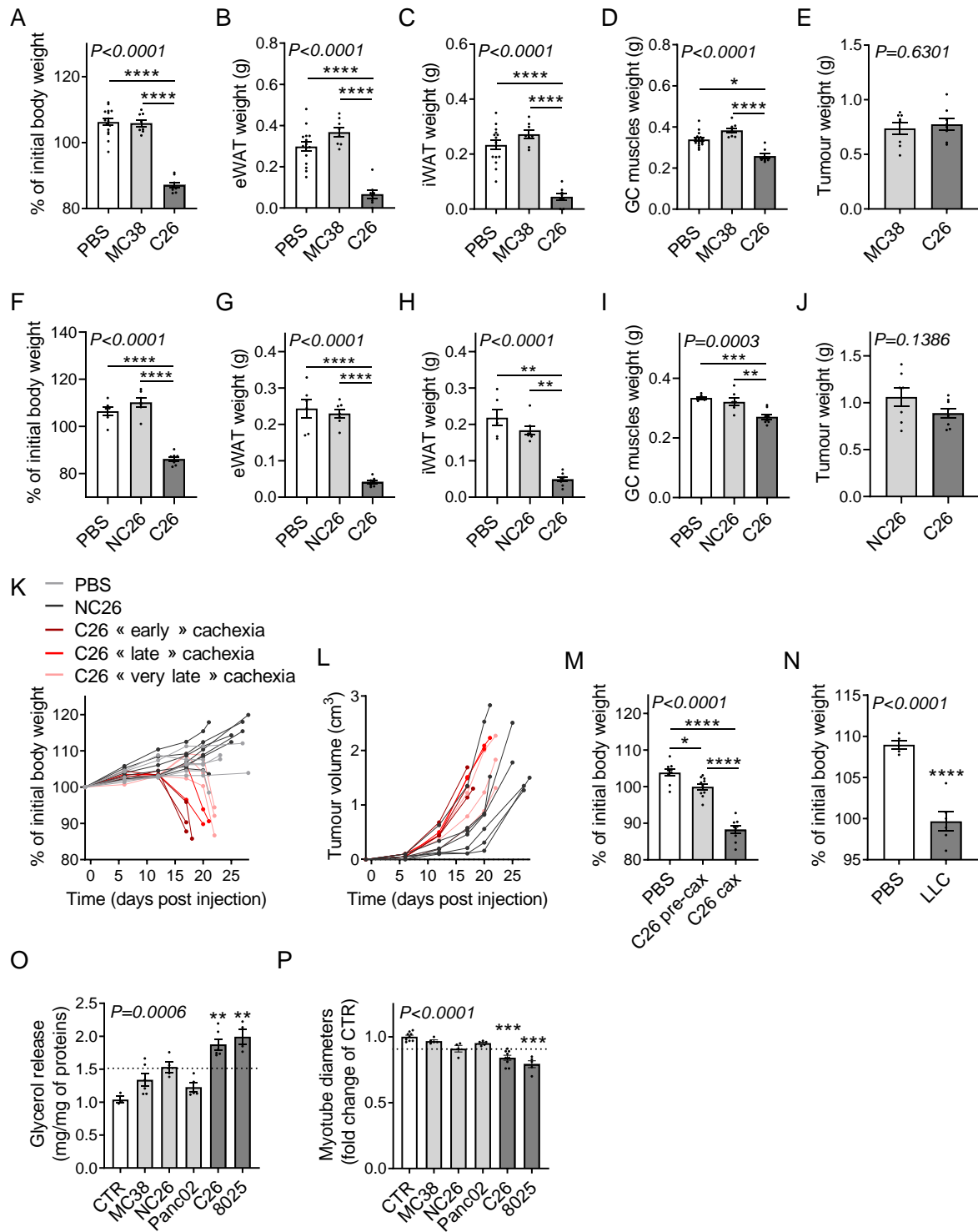

Figure S1

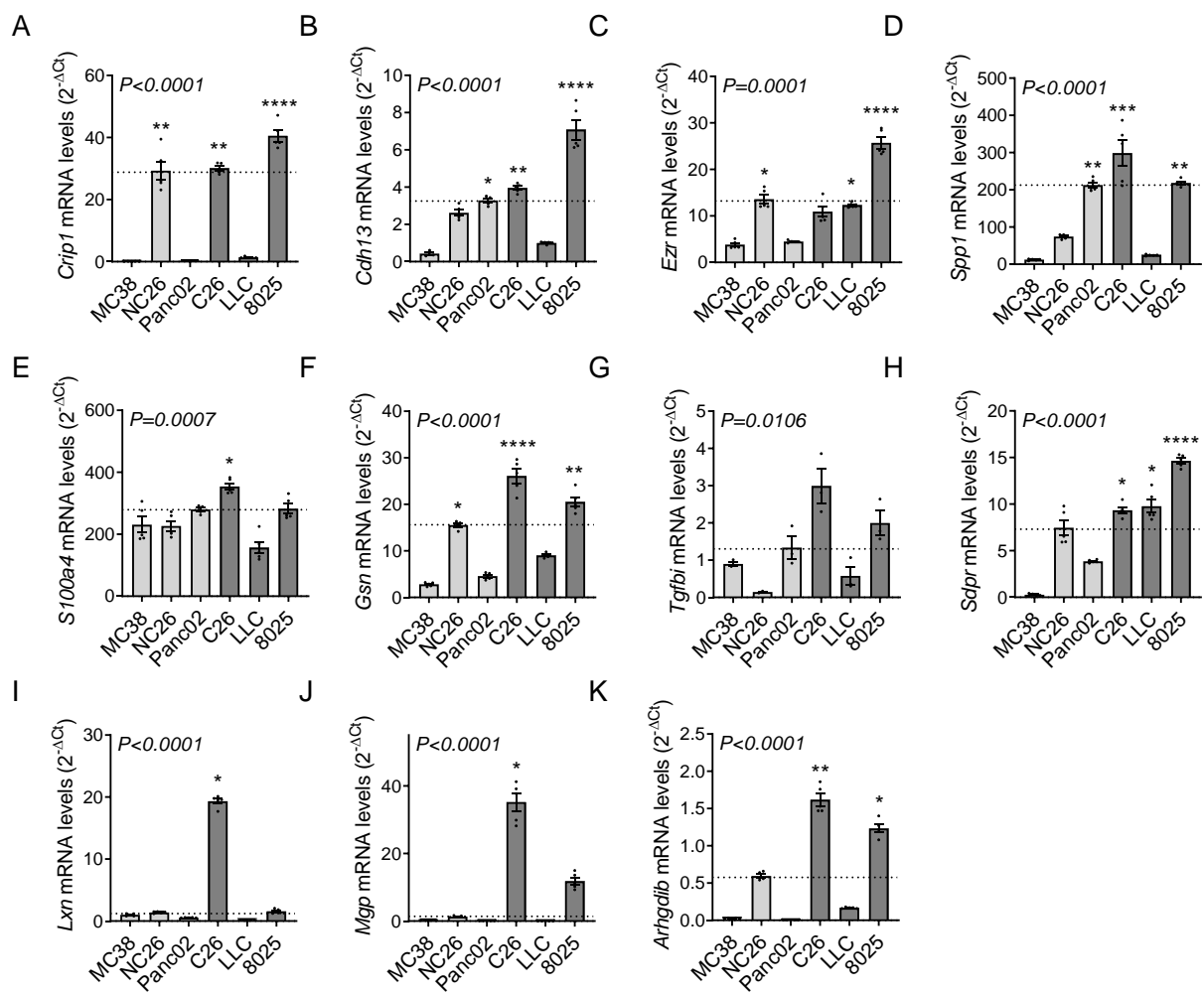

Figure S2

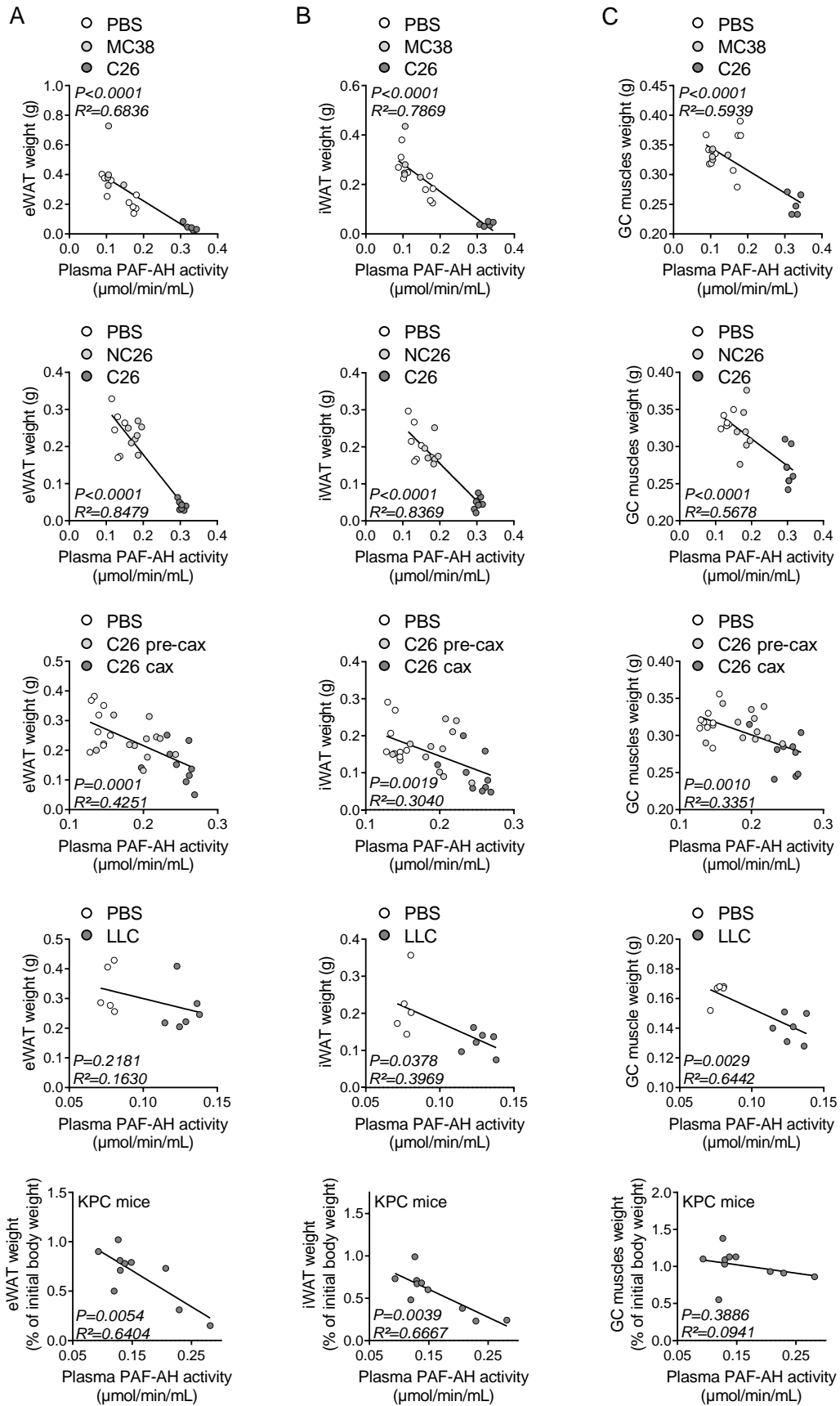

Figure S3

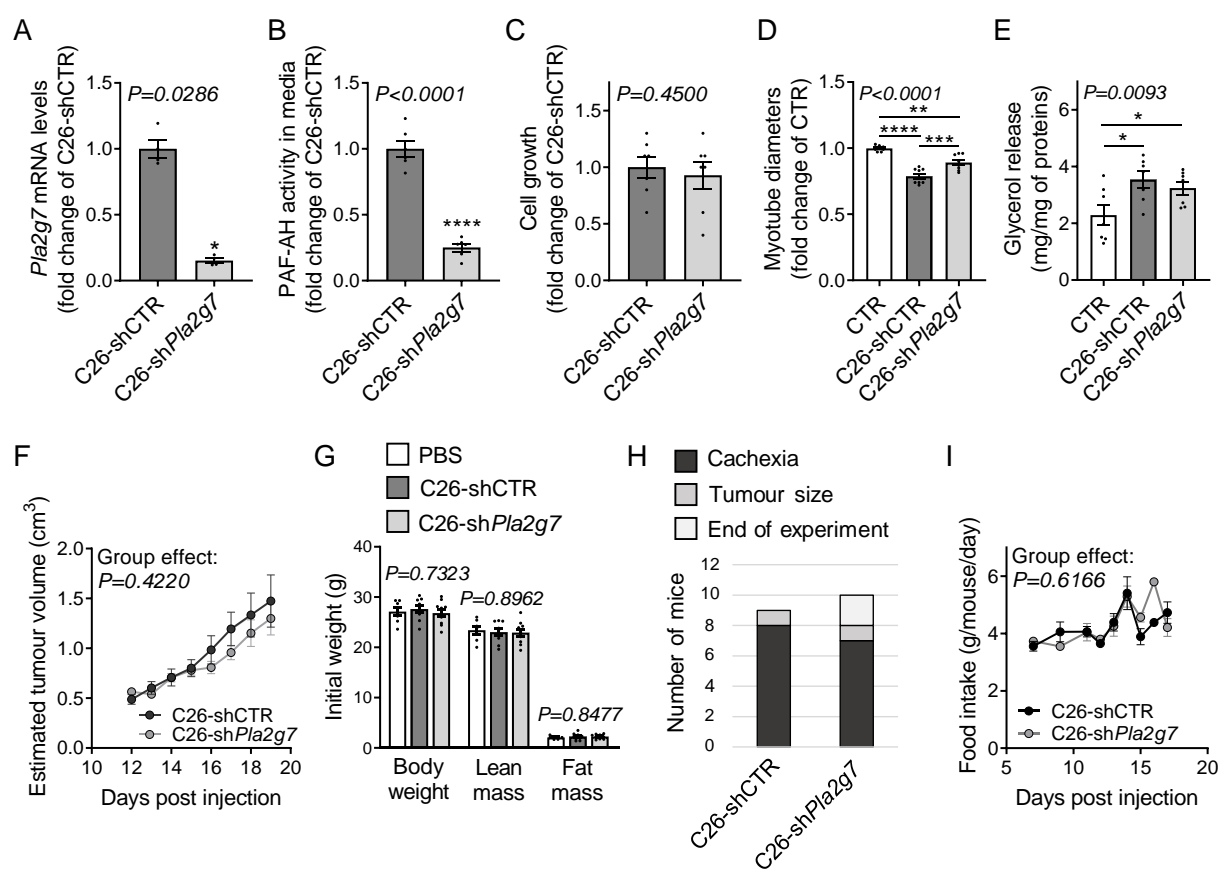

Figure S4

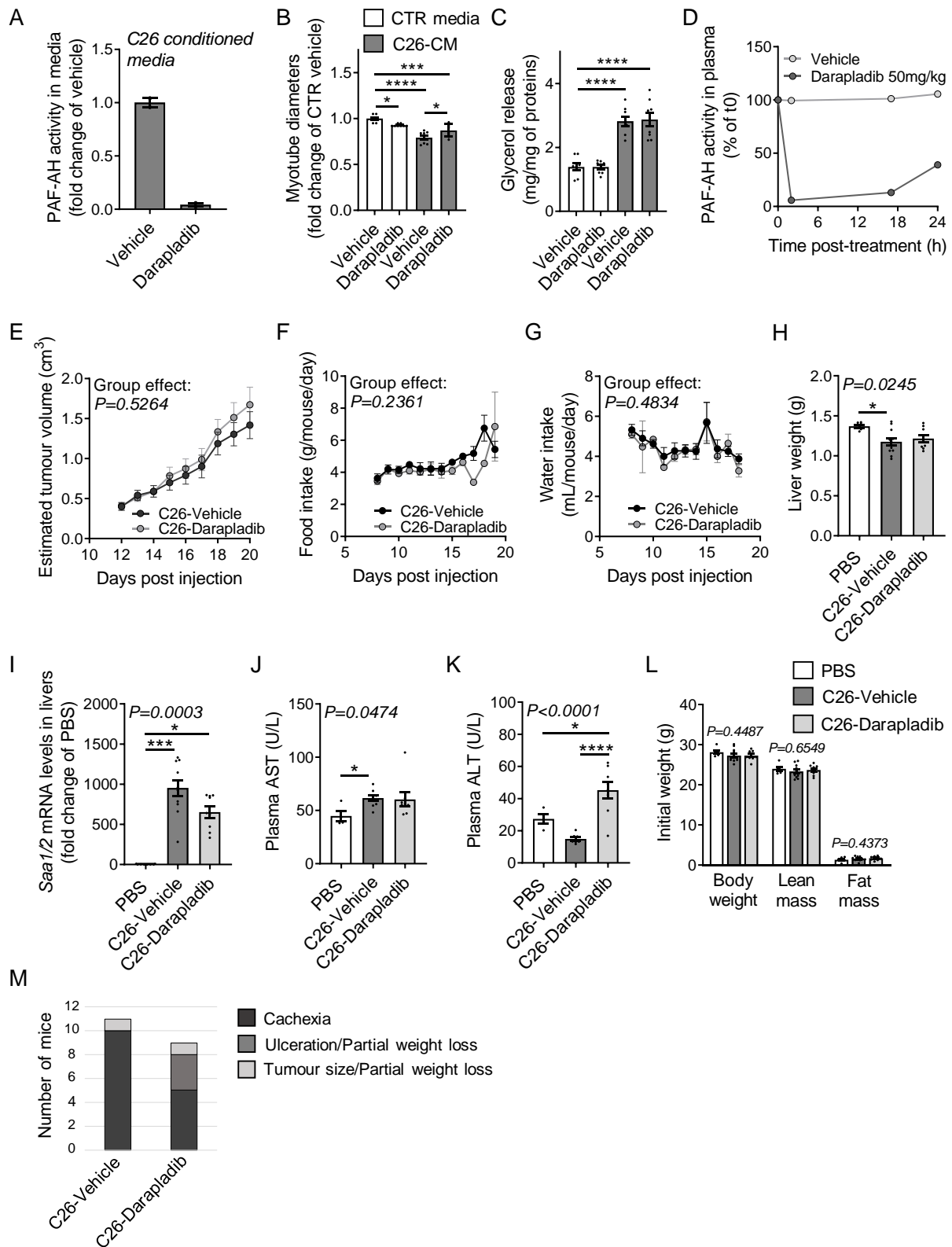

Figure S5

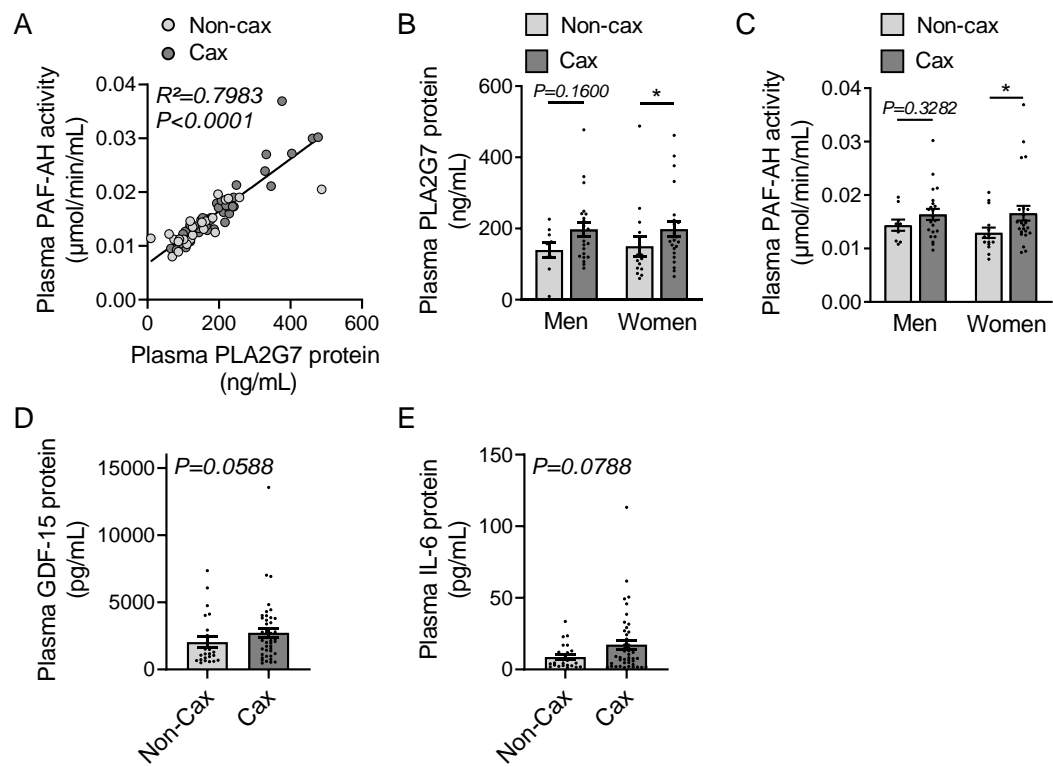

Figure S6

Supplement: Supplementary file 2 — Figure S1. (A‐E) Body weight loss (expressed as percentage of initial body weight) (A), epididymal (eWAT) (B) and inguinal (iWAT) (C) adipose tissues weights, GC muscles (D), and tumours (E) weights of PBS (white bars, n = 16 animals) non‐cachectic MC38 (light grey bars, n = 8 animals) and cachectic C26 (dark grey bars, n = 8 animals) tumour‐bearing mice. (F‐J) Body weight loss (expressed as percentage of initial body weight) (F), epididymal (eWAT) (G) and inguinal (iWAT) (H) adipose tissues weights, GC muscles (I), and tumours (J) weights of PBS (white bars, n = 6 animals) non‐cachectic NC26 (light grey bars, n = 7 animals) and cachectic C26 (dark grey bars, n = 8 animals) tumour‐bearing mice. (K‐L) Longitudinal prospective study showing the evolution of body weight (expressed as percentage of initial body weight) (K) and tumour volume (L) in PBS (light grey lines, n = 6 animals), non‐cachectic NC26 (dark grey lines, n = 7 animals) and cachectic C26 (red lines, n = 8 animals) tumour‐bearing mice throughout cachexia development (mice presented in Figures S1F‐J). C26 mice were divided into 3 groups based on their time course of cachexia development including an early (days 17‐18, dark red lines), late (days 20‐21, bright red lines) and very late (day 22, light red lines) cachexia development. The graphs show individual mice data. (M‐N) Body weight loss (expressed as percentage of initial body weight) of (M) PBS (white bar, n = 9 animals), pre‐cachectic (C26‐precax, light grey bar, n = 11 animals) and cachectic (C26‐cax, dark grey bar, n = 9 animals) C26 tumour‐bearing mice; and (N) PBS (white bar, n = 5 animals) and LLC tumour‐bearing mice (dark grey bar, n = 6 animals). (O‐P) Glycerol released in media of 3T3‐L1 adipocytes (n = 3‐6 biological replicates per group) (O) and diameters of C2C12 myotubes (n = 4‐9 biological replicates per group) (P) treated for 48 hours with normal media (control, CTR, white bars) or conditioned‐media from various cancer cell lines (ligh [file JCSM-12-1333-s003.pdf]
