# Supplementary material for: Association of circulating PLA2G7 levels with cancer cachexia and assessment of darapladib as a therapy
Source: J Cachexia Sarcopenia Muscle. 2021 Aug 23;12(5):1333–51. doi: 10.1002/jcsm.12758 (PMC8517355; doi:10.1002/jcsm.12758)
Supplement: Supplementary file 3 — Supplementary Material Legends [file JCSM-12-1333-s001.docx]

**Supplementary figure legends**

**Table S1.** Proteins differentially secreted in C26 versus MC38 conditioned media with a Log2 value of fold change (FC) > 2 and an adjusted P value > 0.05. n=3 independent biological replicates per group.

**Table S2.** Plasma parameters of mice injected either with PBS (control mice, n=4 animals), control C26 cancer cells (C26-shCTR, n=8 animals) or C26 cancer cells knocked down for Pla2g7 (C26-shPla2g7, n=8 animals). Data are mean ± standard error of the mean. Statistical analyses were performed using unpaired one-way ANOVA with Bonferroni post-hoc tests. Tests were two-sided. * versus PBS. *p<0.05, **p<0.01, ***p<0.001.

**Table S3.** Plasma parameters of mice injected either with PBS (control mice) or C26 cancer cells, and treated once daily either with vehicle (PBS mice n=4 animals, C26-Vehicle tumour-bearing mice n=8 animals) or 50mg/kg darapladib (C26-darapladib tumour-bearing mice n=8 animals). Data are mean ± standard error of the mean. Statistical analyses were performed using unpaired one-way ANOVA with Bonferroni post-hoc tests. Tests were two-sided. * versus PBS. *p<0.05, **p<0.01, ***p<0.001, ****p<0.0001. NS = non-significant.

**Figure S1.**

(A-E) Body weight loss (expressed as percentage of initial body weight) (A), epididymal (eWAT) (B) and inguinal (iWAT) (C) adipose tissues weights, GC muscles (D), and tumours (E) weights of PBS (white bars, n=16 animals) non-cachectic MC38 (light grey bars, n=8 animals) and cachectic C26 (dark grey bars, n=8 animals) tumour-bearing mice.

(F-J) Body weight loss (expressed as percentage of initial body weight) (F), epididymal (eWAT) (G) and inguinal (iWAT) (H) adipose tissues weights, GC muscles (I), and tumours (J) weights of PBS (white bars, n=6 animals) non-cachectic NC26 (light grey bars, n=7 animals) and cachectic C26 (dark grey bars, n=8 animals) tumour-bearing mice.

(K-L) Longitudinal prospective study showing the evolution of body weight (expressed as percentage of initial body weight) (K) and tumour volume (L) in PBS (light grey lines, n=6 animals), non-cachectic NC26 (dark grey lines, n=7 animals) and cachectic C26 (red lines, n=8 animals) tumour-bearing mice throughout cachexia development (**mice presented in Figures S1F-J**). C26 mice were divided into 3 groups based on their time course of cachexia development including an early (days 17-18, dark red lines), late (days 20-21, bright red lines) and very late (day 22, light red lines) cachexia development. The graphs show individual mice data.

(M-N) Body weight loss (expressed as percentage of initial body weight) of (M) PBS (white bar, n=9 animals), pre-cachectic (C26-precax, light grey bar, n=11 animals) and cachectic (C26-cax, dark grey bar, n=9 animals) C26 tumour-bearing mice; and (N) PBS (white bar, n=5 animals) and LLC tumour-bearing mice (dark grey bar, n=6 animals).

(O-P) Glycerol released in media of 3T3-L1 adipocytes (n=3-6 biological replicates per group) (O) and diameters of C2C12 myotubes (n=4-9 biological replicates per group) (P) treated for 48 hours with normal media (control, CTR, white bars) or conditioned-media from various cancer cell lines (light grey bars: no cachexia-inducing properties; dark grey bars: cachexia-inducing properties).

Data are mean ± standard error of the mean. Statistical analyses were performed using one-way ANOVA or Kruskal-Wallis with Bonferroni or Dunn’s post-hoc tests (A-D, F-I, M, O-P) and unpaired t test (E, J, N) respectively. Tests were two-sided. *p<0.05, **p<0.01, ***p<0.001, ****p<0.0001. * *versus* CTR (O-P).

**Figure S2.**

(A-K) mRNA levels of (A) *Crip1*, (B), (B) *Cdh13*, (C) *Ezr*, (D) *Spp1*, (E) *S100a4*, (F) *Gsn*, (G) *Tgfbi*, (H) *Sdpr*, (I) *Lxn*, (J) *Mgp* and (K) *Arhgdib* in various cancer cell lines with different cachexia-inducing properties (n=3-5 independent biological replicates per group; light grey bars: no cachexia-inducing properties; dark grey bars: cachexia-inducing properties). MC38/NC26/C26 are murine colon carcinoma cell lines; Panc02/8025 are murine pancreatic carcinoma cell lines. Data are mean ± standard error of the mean. Statistical analysis was performed using Kruskal Wallis with Dunn’s post-hoc tests. Tests were two-sided. *p<0.05, **p<0.01, ***p<0.001, ****p<0.0001.

**Figure S3.**

(A-C) Linear regression analyses comparing plasma PAF-AH activity and loss of epididymal adipose tissue (eWAT) (A), inguinal adipose tissue (iWAT) (B) and GC muscles mass (C) in (from top to bottom): PBS (white dots, n=10 animals), non-cachectic MC38 (light grey dots, n=5 animals) and cachectic C26 (dark grey dots, n=5 animals) tumour-bearing mice; PBS (white dots, n=6 animals), non-cachectic NC26 (light grey dots, n=7 animals) and cachectic C26 (dark grey dots, n=8 animals) tumour-bearing mice; PBS (white dots, n=9 animals), pre-cachectic (C26-precax, light grey dots, n=11 animals) and cachectic (C26-cax, dark grey dots, n=9 animals) C26 tumour-bearing mice; PBS (white dots, n=5 animals) and LLC tumour-bearing mice (dark grey dots, n=6 animals); and KPC mice with various degrees of body weight loss (n=10 animals). Of note, data from MC38 and C26 tumour-bearing mice are from another cohort than the one presented in **Figure S1A-E** but which shared the same properties (in terms of loss of body weight, fat and muscle mass for a similar tumour size).

Statistical analyses were performed using linear regression analysis.

**Figure S4.**

(A-C) *Pla2g7* mRNA levels (n=4 biological replicates per group) (A), PAF-AH activity in conditioned media (n=6 biological replicates per group) (B), and growth rate (n=7 biological replicates per group) (C) of control C26 cancer cells (C26-shCTR, dark grey bars) and C26 cancer cells stably knocked down for *Pla2g7* (C26-sh*Pla2g7*, light grey bars).

(D-E) Diameters of C2C12 myotubes (n=9 biological replicates per group) (D) and glycerol released in media of 3T3-L1 adipocytes (n=7 biological replicates per group) (E) treated for 48 hours with normal media (control, CTR, white bars) or conditioned media from C26-shCTR (dark grey bars) and C26-sh*Pla2g7* (light grey bars) cancer cells.

(F-I) Mice were injected either with PBS (control mice, white bars, n=7 animals), control C26 cancer cells (C26-shCTR, dark grey bars/lines, n=9 animals) or C26 cancer cells stably knocked down for *Pla2g7* (C26-sh*Pla2g7*, light grey bars/lines, n=10 animals).

(F) Tumour growth curve.

(G) Initial body weight, lean and fat mass (i.e. at the time of cancer cells injection).

(H) Reasons dictating the endpoint at which mice were sacrificed. 8 C26-shCTR mice and 7 C26-sh*Pla2g7* mice developed cachexia. 1 mouse per group was sacrificed because of tumour size reaching humane endpoint before developing cachexia (-2.1% of body weight loss for the C26-shCTR mouse and -2.8% for the C26-sh*Pla2g7* mouse). The two last C26-sh*Pla2g7* mice were sacrificed when the last mouse from the C26-shCTR group developed cachexia. This refers to “end of experiment”.

(I) Food intake over time post cancer cells injection.

Data are mean ± standard error of the mean. Statistical analyses were performed using unpaired (A-B) or paired (C) *t* tests, paired (D-E) or unpaired (G) one-way ANOVA with Bonferroni post-hoc tests, and paired two-way ANOVA (F, I). Tests were two-sided. *p<0.05, **p<0.01, ***p<0.001, ****p<0.0001.

**Figure S5.**

(A) PAF-AH activity in C26 conditioned media supplemented with vehicle or 1µM darapladib (n=2 biological replicates per group).

(B-C) Diameters of C2C12 myotubes (n=8 biological replicates per group) (B) and (C) glycerol released in media of 3T3L1 adipocytes (n=8 biological replicates per group) treated for 48 hours with normal media (control, white bars) or C26 conditioned media (grey bars) supplemented with vehicle or 1µM darapladib.

(D) Plasma PAF-AH activity over 24 hours in mice treated either with vehicle (n=1 animal) or a single dose of 50mg/kg darapladib (n=2 animals).

(E-M) Mice were injected either with PBS (control mice) or C26 cancer cells, and treated once daily either with vehicle (PBS mice, white bars, n=6 animals; C26-Vehicle tumour-bearing mice, dark grey bars/lines, n=11 animals) or 50mg/kg darapladib (C26-Darapladib tumour-bearing mice, light grey bars/lines, n=9 animals).

(E) Tumour growth curve.

(F-G) Food (F) and water (G) intakes over time post cancer cell injection.

(H-I) Liver weights (H) and liver *Saa1/2* mRNA levels (I).

(J-K) Plasma AST (J) and ALT (K) levels.

(L) Initial body weight, lean and fat mass (i.e. at the time of cancer cells injection).

(M) Reasons dictating the endpoint at which mice were sacrificed. 10 C26-Vehicle mice and 5 C26-Darapladib mice developed cachexia. 1 mouse per group was sacrificed because of tumour size reaching humane endpoint but already started to lose weight (-8.5% body weight loss for the C26-Vehicle mouse and -8% for the C26-Darapladib mouse). 3 mice from the darapladib group were sacrificed because of tumour ulceration but already started to lose weight (-4,3% ± 2% body weight loss).

Data are mean ± standard error of the mean. Statistical analyses were performed using paired one-way ANOVA with Bonferroni post-hoc tests (B-C), paired two-way ANOVA (E-G), unpaired one-way ANOVA or Kruskal Wallis with Bonferroni or Dunn’s (H-L) post-hoc tests respectively. Tests were two-sided. *p<0.05, **p<0.01, ***p<0.001, ****p<0.0001.

**Figure S6.**

(A) Linear regression analysis comparing plasma PLA2G7 protein and PAF-AH activity levels in non-cachectic (Non-cax, light grey dots, n=24 individuals) and cachectic (Cax, dark grey dots, n=46 individuals) patients with pancreatic cancer (cohort 2).

(B-C) Plasma PLA2G7 protein (B) and PAF-AH activity (C) levels in non-cachectic (Non-cax, light grey bars, n=24 individuals) and cachectic (Cax, dark grey bars, n=46 individuals) patients classified by gender (cohort 2).

(D-E) Circulating levels of growth differentiation factor 15 (GDF-15) (D) and interleukin 6 (IL-6) in non-cachectic (Non-cax, light grey dots, n=24 individuals) and cachectic (Cax, dark grey dots, n=46 individuals) patients (cohort 2).

Data are mean ± standard error of the mean. Statistical analyses were performed using linear regression (A) and Mann-Whitney test (B-E). Tests were two-sided. *p<0.05.
